# Conserved methylation signatures associate with the tumor immune microenvironment and immunotherapy response

**DOI:** 10.1186/s13073-024-01318-3

**Published:** 2024-04-02

**Authors:** Qingqing Qin, Ying Zhou, Jintao Guo, Qinwei Chen, Weiwei Tang, Yuchen Li, Jun You, Qiyuan Li

**Affiliations:** 1 Department of Hematology, The First Affiliated Hospital of Xiamen University and Institute of Hematology, School of Medicine, Xiamen University, Xiamen, 361003 China; 2 School of Medicine, National Institute for Data Science in Health and Medicine, Xiamen University, Xiamen, 361102 China; 3 Department of Pediatrics, Women and Children’s Hospital, School of Medicine, Xiamen University, Xiamen, 361003 China; 4 Department of Medical Oncology, School of Medicine, The First Affiliated Hospital of Xiamen University and Institute of Hematology, Xiamen University, Xiamen, 361003 China; 5 Xiamen Key Laboratory of Antitumor Drug Transformation Research, The First Affiliated Hospital of Xiamen University, School of Medicine, Xiamen University, The School of Clinical Medicine of Fujian, Medical University, Xiamen, 361003 China; 6 Department of Gastrointestinal Oncology Surgery, The First Affiliated Hospital of Xiamen University, Cancer Center, Xiamen, 361003 China

**Keywords:** DNA methylation signature, Determinant genes, DNA-binding motifs, Immunotherapy response

## Abstract

**Background:**

Aberrant DNA methylation is a major characteristic of cancer genomes. It remains unclear which biological processes determine epigenetic reprogramming and how these processes influence the variants in the cancer methylome, which can further impact cancer phenotypes.

**Methods:**

We performed pairwise permutations of 381,900 loci in 569 paired DNA methylation profiles of cancer tissue and matched normal tissue from The Cancer Genome Atlas (TCGA) and defined conserved differentially methylated positions (DMPs) based on the resulting null distribution. Then, we derived independent methylation signatures from 2,465 cancer-only methylation profiles from the TCGA and 241 cell line-based methylation profiles from the Genomics of Drug Sensitivity in Cancer (GDSC) cohort using nonnegative matrix factorization (NMF). We correlated DNA methylation signatures with various clinical and biological features, including age, survival, cancer stage, tumor immune microenvironment factors, and immunotherapy response. We inferred the determinant genes of these methylation signatures by integrating genomic and transcriptomic data and evaluated the impact of these signatures on cancer phenotypes in independent bulk and single-cell RNA/methylome cohorts.

**Results:**

We identified 7,364 differentially methylated positions (2,969 Hyper-DMPs and 4,395 Hypo-DMPs) in nine cancer types from the TCGA. We subsequently retrieved three highly conserved, independent methylation signatures (Hyper-MS1, Hypo-MS1, and Hypo-MS4) from cancer tissues and cell lines based on these Hyper and Hypo-DMPs. Our data suggested that Hypo-MS4 activity predicts poor survival and is associated with immunotherapy response and distant tumor metastasis, and Hypo-MS4 activity is related to TP53 mutation and FOXA1 binding specificity. In addition, we demonstrated a correlation between the activities of Hypo-MS4 in cancer cells and the fractions of regulatory CD4 + T cells with the expression levels of immunological genes in the tumor immune microenvironment.

**Conclusions:**

Our findings demonstrated that the methylation signatures of distinct biological processes are associated with immune activity in the cancer microenvironment and predict immunotherapy response.

**Supplementary Information:**

The online version contains supplementary material available at 10.1186/s13073-024-01318-3.

## Background

Cancer is characterized by frequent massive alterations in the methylome, which drive a series of major phenotypic changes in cancer cells [1]. Hypermethylation of the promoter selectively inhibits the transcription of tumor suppressors while maintaining chromosomal stability [2–4]. On the other hand, hypomethylation occurs globally in the cancer genome and plays a critical role in oncogenesis and disease development [5, 6]. Differentially methylated positions (DMPs) in specific genes, such as SEPT9, are used as markers for screening prostate and colorectal cancers [7, 8]. Other DMPs influence diverse biological processes by regulating the expression of cancer genes. For example, silencing of MLH1 by hypermethylation of the promoter causes mismatch repair deficiency in colorectal cancer [9]. In addition, patterns of the cancer methylome can be used to determine subtypes of cancers with distinct tissue or cell origins [10–12], pathological characteristics [13], and clinical outcomes [14].

DNA methylation or demethylation in cancer is regulated by DNA methyltransferases (DMNTs), which include DMNT1, DMNT3A, and DMNT3B, and the ten-eleven translocation (TET) enzymes TET1, TET2, and TET3, which are known for their roles in cancer [15]. Nevertheless, the variations in the cancer methylome are more complex than the variations in the activities of methyltransferases. Genetic variants act as mQTLs that alter the methylation levels of CpG loci in cis, thereby influencing gene expression [16, 17]. Histone modifications, such as histone methylation and acetylation, compete with DNA methyltransferase activity to change the landscape of the cancer methylome [18]. Moreover, changes in the cancer methylome are coupled with somatic mutations in cancer driver genes, such as CTNNB1, ERBB2, KRAS, and PIK3CA [16, 19–21], which consistently recapitulate the somatic evolutionary process and consequential clonality of cancers. A recent study has shown that genome and epigenome diversity in prostate cancer can be explained by a unified evolutionary process [22]. In brain tumors, genetic and epigenetic variants act in concert to deregulate the G1/S checkpoint, thereby influencing tumor evolution [23]. Taken together, DNA methylation, along with somatic mutations and gene expression, is a surrogate for critical functional alterations that drive heterogeneous phenotypes of cancer. Many distinct signatures of cancer gene expression and somatic mutations are derived to represent important biological processes in cancer [24, 25]. These signatures are either based on correlations with certain phenotypes of cancer or unsupervised decomposition of parallel, high-throughput profiling of the cancer population [26]. In particular, the latter has yielded many predictive measurements for cancer with strong clinical implications [27]. In addition, methylomic features are increasingly frequently integrated with genomic, epigenomic, and transcriptomic data to predict critical cancer phenotypes [23, 28].

However, the biological processes that shape the landscape of the methylome in cancer cells remain unclear. To date, there have been few efforts to systematically reveal the underlying drivers of the global landscape of the cancer methylome [12]. In addition to technical challenges, cancer methylome profiling is heavily confounded by tumor tissue heterogeneity, sampling biases, and contamination. Moreover, the statistical methods used to identify changes in methylation levels are not as sensitive as those used to identify mutations [29]. In addition, the functional impacts of methylation variations are usually complex [30], which adds to the difficulty in identifying driver genes.

Here, we describe a method for deriving ten independent methylation signatures at the pan-cancer level based on nonparametric statistics and nonnegative matrix factorization (NMF). The resultant methylation signatures showed distinct biological characteristics, which is consistent with the findings of previous studies [12]. Moreover, our findings revealed the endogenous and exogenous drivers of cancer methylome with strong impacts on the vital clinical phenotypes of cancer.

## Methods

### Data sources

We obtained methylation profiles (*n* = 2,465) of nine cancer types from The Cancer Genome Atlas (TCGA), which contains 485,577 CpG site-targeted probes (Illumina Infinium 450 K methylation microarray data). We also obtained matched RNA-seq data (*n* = 2,465), whole-exome sequencing data (*n* = 2,465), and corresponding clinical data (including sex, age, tumor stage, survival time, and tumor purity), representing 2,465 samples of 9 tumor types in the TCGA. In addition, we collected multiomics data from 241 cancer cell lines from the same 9 cancer types from the Genomics of Drug Sensitivity in Cancer (GDSC) [31] dataset of DNA methylation data (Illumina HumanMethylation450 BeadChip). We also collected protein–DNA- binding site data with TF-DNA-seq from the ENCODE project.

### Sample purity filtering and probe quality control

We selected tumor samples with a purity greater than 0.6 (Additional file 2: Table S1). Subsequently, the 450 K microarray data probes were filtered according to the following steps. First, locations containing SNPs were removed; second, probes on chromosomes X and Y were filtered; and finally, probes with more than 10% missing values were filtered. Ultimately, the remaining 381,900 probes containing missing values were imputed with all samples estimated median *β* values.

### Deriving differentially methylated positions (DMPs)

To obtain conserved Hyper and Hypo-DMPs in tumors, we performed 1000 rounds of permutation tests of 381,900 probes in the methylation matrix of 569 pairs of samples from nine cancer types (i.e., shuffling the matched tumor and normal probes within each pair). Consequently, we obtained the null distribution of *β* via the R package “predictmeans.” We used the tissue’s origin as a covariate in the model and selected significant probes (*P* < 0.001) as poised DMPs. The function is as follows:

$${\beta }_{ij}={\beta }_{0}+{\beta }_{1}\times {TY}_{i}+{\beta }_{2}\times {CY}_{ik}+{\beta }_{3}\times ({TY}_{i}:{CY}_{i})+{TO}_{i}+{\varepsilon }_{ij}$$

In this model, ${\beta }_{ij}$ represents the methylation level (ranging from 0 to 1) of the ${j}_{{\text{th}}}$ probe in the *i*_th_ sample, ${TY}_{i}$ represents the tissue type (either cancerous or adjacent noncancerous) of the ${i}_{{\text{th}}}$ sample, ${CY}_{ik}$ represents the ${k}_{{\text{th}}}$ cancer type in the ${i}_{{\text{th}}}$ sample, and ${TO}_{i}$ represents the origin of the tissue for the ${i}_{{\text{th}}}$ sample. ${\beta }_{0}$ is the intercept,${\beta }_{1}$ is the coefficient pertaining to the tissue type, ${\beta }_{2}$ is the coefficient for the cancer type, and ${\beta }_{3}$ represents the coefficient for the interaction between the tissue type (cancerous and adjacent noncancerous) and the type of cancer. Finally, ${\varepsilon }_{ij}$ refers to the Gaussian error term.

Next, we calculated “delta-beta” ($\Delta \beta$) for each probe by subtracting the *β* value of the tumor-adjacent normal tissue from the *β* value of the matched tumor tissue. Then, we calculated the median $\Delta \beta$ values as the expected difference in methylation level between tumors and adjacent normal tissues, $\Delta \beta$. We subsequently classified the poised DMPs into poised Hyper-DMPs ($\Delta \beta$> 0) and poised Hypo-DMPs ($\Delta \beta$< 0) based on the sign of the estimated difference $\Delta \beta$. To control for unspecific variation in the beta values, we further set a threshold (0.175) for $\Delta \beta$ of Hyper-DMPs based on the 5% extreme of low methylation of the poised Hyper-DMPs (the 5th percentile of the distribution of ∆*β*, − 0.175). Similarly, we set a threshold (− 0.145) for $\Delta \beta$ Hypo-DMPs based on 5% of the extremely high methylation of the poised Hypo-DMPs (0.145) (Additional file 1: Fig. S1).

### Analyzing the binding activities of specific proteins to DMPs

To investigate the relationships between DMPs and DNA methyltransferases and demethylating enzymes, we obtained ChIP-seq data for DNMT3A and DNMT3B (HepG2) from ENCODE and published ChIP-seq data for TET2 (MCF7) [32]. We obtained multiple independent datasets of FOXA1 binding from ChIP-Seq experiments, including four cancer cell lines (A549, HepG2, and MCF-7 from ENCODE and T47D [33]) and tissues (colorectal cancer and breast cancer) [34, 35]. Then, we analyzed the binding activities of these enzymes to Hyper-DMPs and Hypo-DMPs using “deepTools” [36].

### Deriving DNA methylation signatures using nonnegative matrix factorization (NMF)

We applied nonnegative matrix factorization (NMF; R package NMF v0.22.0) to the matrices of 2465 tumor samples containing the hyper-DMPs or hypo-DMPs’ *β* values or 1-*β* values that we obtained above from the permutation test. NMF is an unsupervised, parts-based learning paradigm that decomposes a nonnegative matrix *M* into two matrices.

$${V}_{p\times n}= {E}_{p\times k} \times {H}_{k\times n}$$

*V* is a *p* × *n* DNA methylation matrix, where *p* = 2649 for Hyper-DMPs’ *β* values or *p* = 4395 for Hypo-DMPs’ *β* or 1-*β* values, and *n* = 2465 TCGA samples. *H* is the signature activity matrix of *k* methylation signatures in *n* cancers; matrix *E* is the loading matrix corresponding to the weights of *p* probes in *k* methylation signatures [37]. We denoted each signature’s most highly weighted DMPs as up-DMPs if we used *β* values to derive the signature, while we used the term “down-DMPs” (the DMPs list is deposited at https://github.com/xmbd/Pan_Meth_Sig) if we used 1-*β* values to derive the signatures. We used the cophenetic correlation coefficient and silhouette width to determine the optimal *k* (the number of signatures) corresponding to the most robust clustering [37, 38] for the hyper and hypomethylation matrices, respectively. A preferred *k* (*k* = 3 for the hypermethylation signature and *k* = 7 for the hypomethylation signature) was selected by leveraging the cophenetic coefficient score and average silhouette width. The analysis was performed using the R package NMF. The signature and DMP were estimated by NMF using a clustering method. For each signature, highly weighted DMPs and samples are routinely assigned to a signature by NMF. We used cosine similarity (CS; R package PharmacoGx) and Spearman correlations of DMPs to evaluate the independence or similarity among the methylation signatures.

### Identification of the deterministic genes in the methylation signatures

Instrumental variable (IV) analysis was employed to identify the driver genes affecting the methylation signatures, as previously described [39]. IV analysis was performed using the screened genes via the R package “ivpack” [version 1.2]. We first screened for genes with a mutation status (0 stands for wild type, 1 stands for SNV mutation/frameshift; mutation number > 10) significantly associated with the methylation signature activity by the Wilcoxon rank sum test (FDR < 0.1; Additional file 2: Table S3). We then performed IV analysis using the filtered genes (Additional file 2: Table S3) as inputs. Notably, we defined the genes’ somatic mutational status (missense or frameshift) as an instrumental variable, gene expression level as an independent variable (mediator), methylation signature as a dependent variable (outcome), and cancer type as a covariate [40].

$${MS}_{ki}={\beta }_{0}+{\beta }_{1}\times {mRNA}_{ij}$$

Here, ${\varepsilon }_{ijk}$~N_0_,σ^2^ is a Gaussian error term; ${MS}_{ki}$ is the ${i}_{{\text{th}}}$ individual’s ${k}_{{\text{th}}}$ methylation signature; mRNA_*ij*_ is the ${i}_{{\text{th}}}$ individual’s ${j}_{{\text{th}}}$ gene expression; ${SMS}_{ij}$ is the somatic mutational status of the ${j}_{{\text{th}}}$ gene of the ${i}_{{\text{th}}}$ individual; and ${CY}_{i}$ is the ${i}_{{\text{th}}}$ individual’s cancer type.

Regression coefficients were estimated using two-stage least squares regression. To determine the significance of the independent variables, we calculated the FDR using the Benjamini–Hochberg method for the regression coefficients based on the *P* values of the Wu–Hausman test. To determine the significance of the instrumental variables, we used the weak instrument test *P* values [41] based on the Kleibergen–Paap rank Wald *F*-statistic and estimated FDR_weak_. Finally, we selected genes whose expression levels and genetic instruments were both significant (FDR _Wu-hausman_ < 0.05, FDR_weak_ < 0.05, and FDR_model_ < 0.05). The detailed code is deposited at https://github.com/xmbd/Pan_Meth_Sig.

### Enrichment of DNA-binding motifs

Enrichment of DNA-binding motifs in the methylation signatures was evaluated using the “findMotifsGenome.pl” module available in HOMER (http://homer.ucsd.edu/homer/ngs/peakMotifs.html) [42]. First, for each DMP in a given methylation signature, we classified the DMPs into two subgroups based on their location, “TSS” or “gene body.” Then, we retrieved the DNA sequences within a 100-bp range of the corresponding 450 k array probe set. We evaluated the enrichment of known DNA binding motifs within the DNA fragments corresponding to DMP sets in certain methylation signatures based on default parameters and reported the motifs with a significance of *P* < 0.01.

### Survival analysis

To evaluate the impact of each methylation signature on the clinical outcome of cancer, we classified the samples into two groups according to the medians of signature activities. Cox proportional hazards regression was used to assess the association of each signature with overall survival (OS) (*survival* R package v2.42.3) in individual cancer types.

We also tested the effect of Hypo-MS4 on overall survival together with the TP53 mutation status and FOXA1 gene expression to adjust for possible confounding effects.

### Prediction of immunotherapy response

To investigate the relationship between Hypo-MS4 activity and ICI response, we downloaded two methylome datasets of melanoma patients with annotated clinical information [43, 44]. We retrieved the methylation matrix (*V*) for the DMPs and subsequently used the weight matrices derived from both tumor tissues (*E*) to calculate each tumor’s methylation signature scores. Finally, we compared the methylation signature activities between the responders and nonresponders using Wilcoxon’s rank sum test.

In addition, we evaluated the differences in gene expression influenced by Hypo-MS4 activity, referred to as Hypo-MS4-GES, between responders and nonresponders in gastric cancer and melanoma [45–47]. We downloaded the single-cell RNA sequences of melanoma patients who received immunotherapy and retrieved the Hypo-MS4-GES score for each cancer cell. We then examined the fraction of cancer cells with high Hypo-MS4-GES scores and compared the scores between responders and nonresponders before and after treatment using Wilcoxon’s rank sum test [48]. In addition, we used a multivariate linear regression model to evaluate the predictive power of the Hypo-MS4-GES score, TP53 mutational status and FOXA1 gene expression in the melanoma dataset [46] as follows:

$${ICI}_{i}={\beta }_{0}+{\beta }_{1}\times {MS4}_{i}+{{\beta }_{2}\times TP53}_{i}+{\beta }_{3}{FOXA1}_{i}+{\varepsilon }_{i}$$

Here, ${\varepsilon }_{i}$~(N_0_,σ^2^) is a Gaussian error term; ${ICI}_{i}$ is the ${i}_{{\text{th}}}$ patient’s ICI response; MS4_*i*_ is the activity of Hypo-MS4 of the ${i}_{{\text{th}}}$ patient; ${TP53}_{i}$ is the TP53 mutational status of the ${i}_{{\text{th}}}$ patient; and ${FOXA1}_{i}$ is the FOXA1 gene expression of the ${i}_{{\text{th}}}$ patient .

### Statistical analyses

All the statistical analyses and data visualization were performed using R (version 4.1.1). The statistical significance of all the statistical tests was set at *P* or adjusted *P* < 0.05 (FDR correction).

## Results

### Deriving DNA methylation signatures across 2465 cancers

**Fig. 1:**
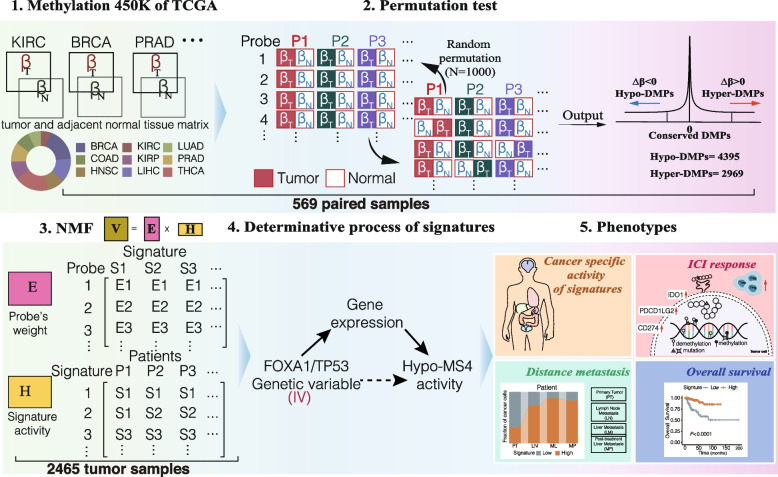
Workflow of deriving DNA methylation signatures in cancers

We constructed a workflow to derive conserved DNA methylation signatures (Fig. 1A). We first analyzed 569 paired methylation profiles of both tumor and matched normal tissues from The Cancer Genome Atlas (TCGA) (Additional file 1: Supplementary Methods). We retrieved 253,574 differentially methylated positions (DMPs) based on the significance of differences in beta values (∆$\beta$) between matched tumor and normal tissue in nine cancer types (permutation test, *P* < 0.001) (Methods, Additional file 1: Fig. S1, Additional file 2: Table S1). Among the significant DMPs, 129,868 were hypermethylated, and 122,782 were hypomethylated in cancer patients. We further selected the most conserved significant Hyper-DMPs (*N* = 2969) and Hypo-DMPs (*N* = 4395) by controlling the variation in ∆$\beta$ (Additional file 1: Supplementary Methods; Additional file 1: Fig. S1). To verify the DMPs, we compared the methylation levels of the top 10 Hyper and Hypo-DMPs between cancer and normal tissues in 9 cancer types and showed that the DMPs were indeed significantly differentially methylated among the cancer types (Additional file 1: Fig. S2 and S3). We characterized the genomic locations of Hyper and Hypo-DMPs and found that most of the Hypo-DMPs (71.07%) were located in the Open Sea region, while the majority of the Hyper-DMPs (67.03%) were located at CpG islands (Additional file 1: Fig. S4A-B), suggesting a general tendency toward reprogramming of the cancer methylome. We then examined the binding activities of known regulators of DNA methylation, namely, DNMT1, DNMT3B, and TET2, at the DMPs [32, 40]. Of the three genes, TET2-binding activity was selectively enriched at Hypo-DMPs in MCF-7 cells (Additional file 1: Fig. S4C), while DNMT1 and DNMT3B-binding activity were not enriched at the DMPs (Additional file 1: Fig. S4D, E).

To further understand the variations in the conserved DMPs in cancer, we applied nonnegative matrix factorization (NMF) to the methylation profiles of Hyper-DMPs (*n* = 2,969) and Hypo-DMPs (*n* = 4,395) in 2,465 TCGA cancer samples representing nine cancer types. As a result, we identified three distinct hypermethylation signatures (hereafter, Hyper-MS1-3) and seven hypomethylation signatures (hereafter, Hypo-MS1-7) (Methods) based on cophenetic coefficients and silhouette coefficients (Additional file 1: Fig. S5A). Each methylation signature was defined as a positive linear combination of a distinct set (321 to 1231) of contributing DMPs. The contributions of each DMP to a given methylation signature (probe weight, E) were all nonnegative and summed to one (Additional file 1: Fig. S5B). For comparison, we also derived hypomethylation signatures using 1-*β* values, namely, Hypo-MSs-down, and found that the resulting signature scores were nearly identical to the original Hypo-MSs derived from *β* values (Methods, Additional file 1: Fig. S6).

### The landscape and characteristics of the DNA methylation signatures

**Fig. 2:**
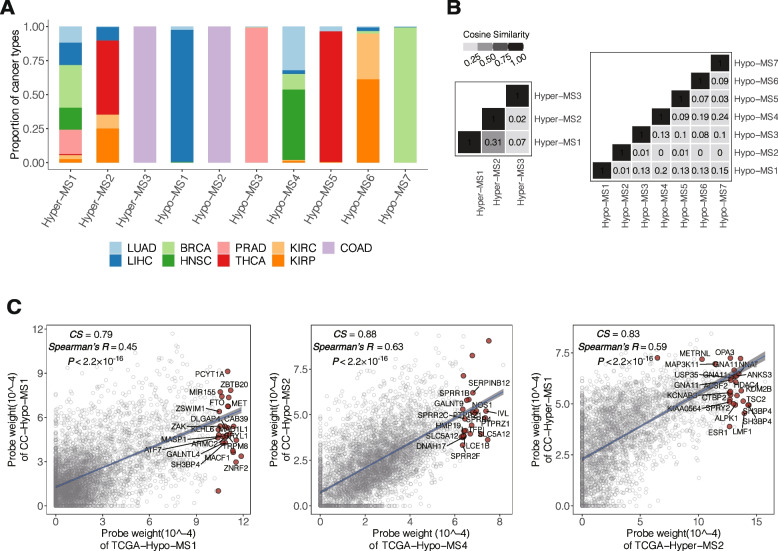
Characterization of DNA methylation signatures in cancers. **A** Proportion of each cancer type in which different DNA methylation signatures are present. **B** Cosine similarity between different methylation signatures (MSs). **C** Cosine Similarity and Spearman correlation between the probe weight of methylation signatures derived from cancer cell lines (CC-MSs) and tumor tissues (TCGA-MSs). The top 20 weighted probes in each signature are labeled with the corresponding gene names.

We found that the activities of DNA methylation signatures are highly variable in the cancer population (Fig. 2A and Additional file 1: Fig. S7A). The majority (78.09% ~ 99.63%) of the variation was attributed to the different tissue origins of cancer (Additional file 2: Table S2; Additional file 1: Supplementary Methods). Our data showed that all cancer types exhibited one to eight predominant methylation signatures. Some methylation signatures are cancer type specific. For example, Hyper-MS3 and Hypo-MS2 are active specifically in COAD, and Hypo-MS3 activity is specific to PRAD. In contrast, several methylation signatures, such as Hypo-MS4 (HNSC, LUAD, LIHC, BRCA, KIRP, and KIRC) and Hyper-MS1 (BRCA, HNSC, LIHC, PRAD, KIRC, KIRP, and THCA) (Fig. 2A), were active in multiple cancer types. In addition, the DMPs contributing to the hyper and hypomethylation signatures exhibited distinct patterns of localization (Additional file 1: Fig. S7B), suggesting a general tendency toward reprogramming of the cancer methylome.

The activities of the Hyper-MSs (cosine similarity, CS ≤ 0.31) and the Hypo-MSs (CS ≤ 0.24) (Fig. 2B and Additional file 1: Fig. S7C) in the TCGA population were highly independent, suggesting that these signatures represent distinct biological activities. To further verify the conservativity and consistency of the methylation signatures derived from heterogeneous tumor tissues, we performed NMF on the methylation profiles of the same set of DMPs used to derive the hyper and hypomethylation signatures in 241 cancer cell lines representing the same nine cancer types (Additional file 1: Supplementary Methods). We obtained four cancer cell-based hyper and hypomethylation signatures (CC-Hyper-MS1 and 2, CC-Hypo-MS1 and 2) (Additional file 1, Fig. S7D). We evaluated the consistency of the probe-weight matrix E between cell-based methylation signatures and tissue-based signatures (Additional file 1: Supplementary Methods). The TCGA-derived Hypo-MS1 (tumor tissue) was highly consistent with CC-Hypo-MS1 (CS = 0.79, Spearman’s *R* = 0.45) and TCGA-Hypo-MS4 consistent with CC-Hypo-MS2 (CS = 0.88, Spearman’s *R* = 0.63). In addition, TCGA-Hyper-MS2 resembled CC-Hyper-MS1 (CS = 0.83, Spearman’s *R* = 0.59) (Fig. 2C). Among the most conserved and highly weighted genes between the cell-based and tumor methylation signatures were many known oncogenes, such as ETS1, RORA, and CDKN1A (Hypo-MS1); immune genes, such as SIGLEC9, SIGLEC7, MUC15, and LAIR1 (Hypo-MS4); and SEPT9 [49] and DDIT3 [50] (methylation-related biomarkers) for Hyper-MS2. These findings suggest that Hyper-MS2, Hypo-MS1 and Hypo-MS4 are driven by conserved biological processes in cancers.

### DNA methylation signatures are associated with clinical features

Many previous studious have shown the methylome correlates with many clinical and pathological features [51, 52]. Therefore, we verified the relationship between our methylation signatures and clinical outcomes. We divided the patients into two groups (low and high) according to the median methylation signature activity and then investigated the associations among the groups and patient age, overall survival, and cancer stage within individual cancer types (Methods). As a result, most of the Hypo-MSs (6/7) showed no association with patient age in the cancer types where they were present, except for Hypo-MS5, which was negatively associated with age in the THCA (FDR = 0.00738) (Wilcoxon rank-sum test). For the Hyper-MSs, Hyper-MS1 and Hyper-MS2 exhibited significant correlations with age in BRCA and THCA (Hyper-MS1: FDR_BRCA_ = 0.00198, FDR_THCA_ = $3.96\times {10}^{-16}$; Hyper-MS2: FDR_BRCA_ = 0.00396, FDR_THCA_ = $2.43\times {10}^{-7}$; Additional file 1: Fig. S8A, B).

**Fig. 3:**
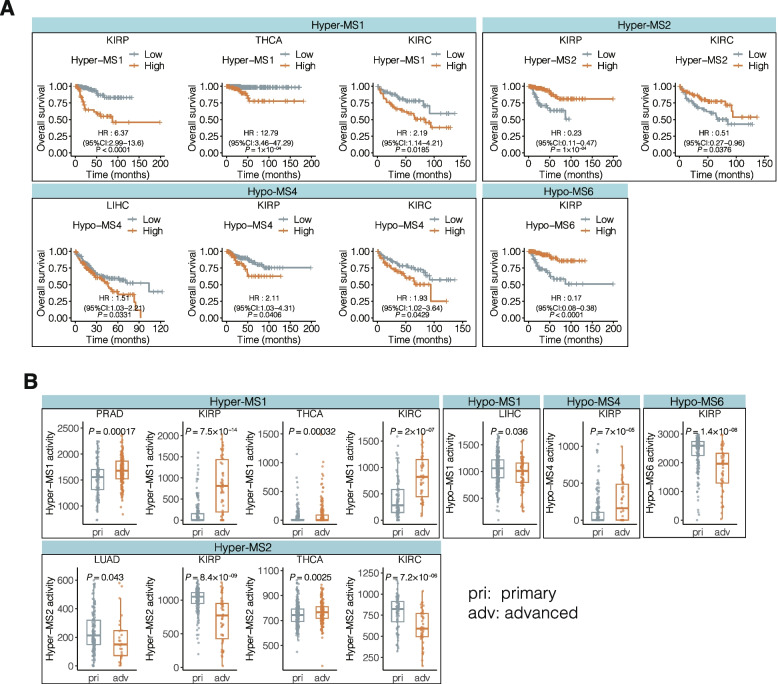
Methylation signature activity associated with disease outcome. **A** Survival analysis for patients with different DNA methylation signatures was conducted for the individual cancer types in which the corresponding signatures were present. Patients were stratified into two groups based on median signature activity. **B** Relationships between cancer stage and the activity of methylation signatures in individual cancer types, where the corresponding methylation signatures are present. Patients were stratified into two groups according to tumor stage (stage I, II: pri.; stage III, IV: adv.)

Next, we examined the signature’s predictive power for survival. We performed survival analysis using the same patient groups based on signature activities in individual cancer types where the specific signatures were present (Fig. 3A and Additional file 1: Fig. S9A). As a result, we found that Hypo-MS4 was strongly predictive of poor OS in 3 out of the 6 cancer types in which Hypo-MS4 was present, such as LIHC (HR = 1.51, *P* = 0.03), KIRC (HR = 2.11, *P* = 0.04), and KIRP (HR = 1.93, *P* = 0.04) (Fig. 3A). Moreover, Hyper-MS1 predicted better survival in 3 out of the 8 cancer types tested, such as KIRC (HR = 2.19, *P* = 0.02), KIRP (HR = 6.37, *P* < 0.0001), and THCA (HR = 12.79, *P* = 1 $\times {10}^{-4}$) (Fig. 3A). Since Hypo-MS4 is independent of age, our data suggest that Hypo-MS4 is a predictor of poor prognosis in multiple cancer types.

Moreover, half of the methylation signatures (5/10) were related to cancer stage (Fig. 3B and Additional file 1: Fig. S9B). Our data showed that Hyper-MS1, Hyper-MS2, and Hypo-MS4 were significantly associated with advanced (stage III–IV) tumors (*P* < 0.05, Wilcoxon’s rank sum test; Fig. 3B). Among them, Hyper-MS1 was consistently associated with advanced cancer stage in four cancer types tested, namely, PRAD, THCA, KIRC, and KIRP, suggesting a unique role in tumor progression.

Taken together, these findings indicate that methylation signatures are associated with several clinical features. In particular, Hypo-MS4 activity is strongly correlated with poor patient survival, which is independent of age.

### Methylation signatures associated with the tumor immune microenvironment (TIME)

**Fig. 4:**
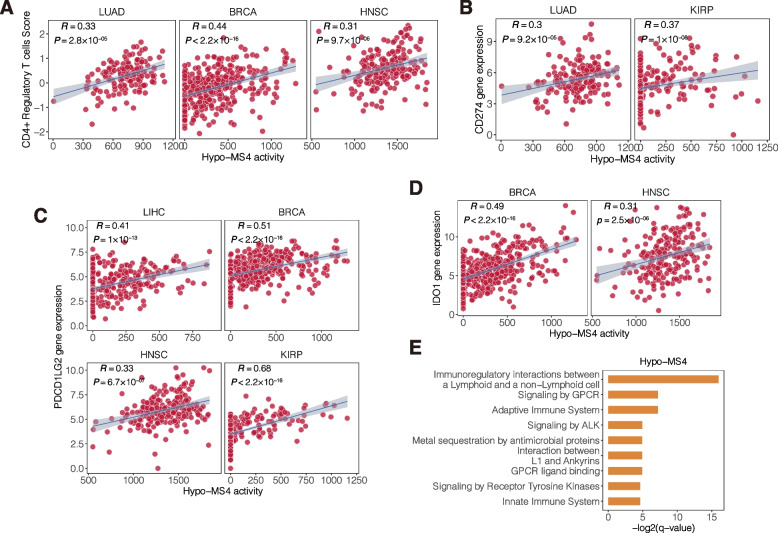
The relationship between hypomethylation signature 4 activity and the tumor immune microenvironment. **A** Correlations between the immune activity and CD4 + regulatory T cell infiltration and Hypo-MS4 activity in LUAD, BRCA and HNSC. **B** Correlation between the gene expression levels of CD274 and Hypo-MS4 activity in LUAD and HNSC. **C** Correlations between the gene expression levels of PDCDELG2 and Hypo-MS4 activity in LIHC, BRCA, HNSC, and KIRP. **D** Correlation between the gene expression levels of IDO1 and Hypo-MS4 activity in BRCA and HNSC. **E** Significantly enriched Reactome pathways of Hypo-MS4-associated genes (*Q* value < 0.05)

As previous studies demonstrated that DNA methylation is associated with cancer immunity [53, 54], we assessed the impacts of methylation signatures on the tumor microenvironment using known lymphocyte activities and immune cell fractions in nine cancer types [55, 56] and calculated the meta-effects (Spearman’s correlation coefficient, Methods) for each signature. Among the ten methylation signatures, four demonstrated significant correlations with the activities of lymphocytes in the tumor microenvironment (FDR < 0.05) (Additional file 1: Fig. S10A). In particular, Hypo-MS4 was strongly positively associated with diverse immune cell activities in most cancer types, such as CD4 + regulatory T cells (Spearman’s *R* > 0.3, *P* $\le$ $2.4\times {10}^{-5}$ in LUAD, BRCA, and HNSC) (Fig. 4A and Additional file 1: Fig. S10C) and lymphocyte infiltration (Spearman’s *R* > 0.3, *P* $\le 1.4\times {10}^{-6}$ in LIHC, BRCA, HNSC, THCA, and KIRP) (Additional file 1: Fig. S10D).

We further examined the correlation between the signature activity and the expression levels of immune-related genes and obtained consistent results for Hypo-MS4, Hypo-MS1, and Hypo-MS7 (Additional file 1, Fig. S10B). In particular, Hypo-MS4 activity was positively associated with the expression levels of immune checkpoint genes, namely, CD274, PDCD1LG2, and IDO1, in multiple cancer types (*P* ≤ 0.01; Fig. 4B-D; Additional file 1: Fig. S10E-G). Moreover, Hypo-MS4-associated genes were strongly enriched in immune pathways (Methods, Fig. 4E).

Taken together, the effects of Hypo-MS4 on the TIME are the most prominent among all the methylation signatures, suggesting its unique role in the interaction between cancer cells and the microenvironment and its potential influence on cancer immunity.

### Hypo-MS4 is associated with tumor immune evasion and the ICI response

We think that the somatic mutation burden or neoantigen load may underlie the association between Hypo-MS4 and cancer immunity. We analyzed the tumor mutational burden (TMB) and neoantigen load in the tumor types in which Hypo-MS4 was present. We observed no significant correlations between TMB and Hypo-MS4 in LUAD, LIHC, and KIRC and a moderate negative association in HNSC and KIRP (*P* < 0.05). In BRCA, TMB was positively associated with Hypo-MS4 (Additional file 1: Fig. S11A, B).

**Fig. 5:**
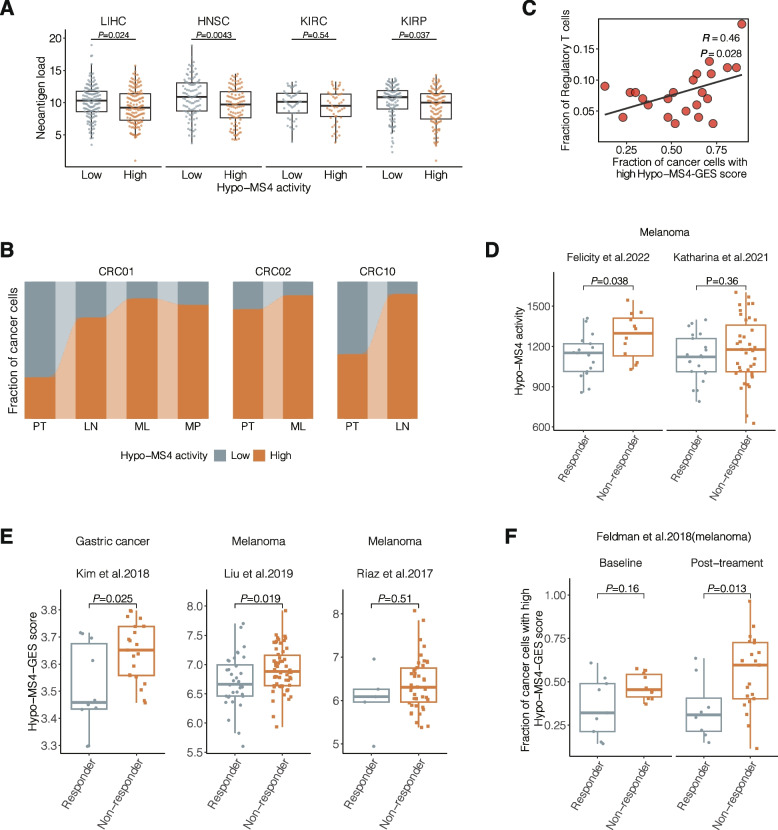
Hypo-MS4 is a signature of tumor immune responses that is associated with distant metastasis. **A** Comparison of neoantigen load between tumors of low and high Hypo-MS4 activity in four cancer types. **B** Correlation analysis of the fraction of cancer cells with high Hypo-MS4 activity and the fraction of CD4 + regulatory T cells in melanoma. **C** Fractions of cancer cells with high Hypo-MS4-GES scores in primary lesions and lymph node metastases from patients with colorectal cancer in single-cell methylome datasets of three patients, CRC01, CRC02, and CRC10. **D** Differences in Hypo-MS4 activity in responders and nonresponders to ICI therapy (Wilcoxon**’**s rank sum test) according to two methylome sequencing studies of melanoma patients. **E** Hypo-MS4-GES score in responders and nonresponders to ICI therapy (Wilcoxon’s rank sum test), in patients with gastric cancer (Kim et al. (2018) [45]) and in patients with melanoma (Liu et al. (2019) [46]; Riaz et al. (2017) [47]). **F** Fraction of Hypo-MS4-positive cells in ICI therapy responders and nonresponders (Wilcoxon’s rank sum test) before and after ICI treatment based on the single-cell transcriptome of melanoma

For the neoantigen load, we found that tumors with high Hypo-MS4 activity showed consistently lower neoantigen loads in 4 out of the 6 cancer types tested, including LIHC (*P* = 0.024), HNSC (*P* = 0.0043), KIRC (*P* = 0.54), and KIRP (*P* = 0.037) (Fig. 5A). In LUAD patients, there was no significant association between neoantigen load and Hypo-MS4 activity (*P* = 0.96; Additional file 1: Fig. S11C). In BRCA, the neoantigen load was positively associated with Hypo-MS4 status (*P* = 0.00031; Additional file 1: Fig. S11D). However, this association with neoantigen load became nonsignificant within BRCA patients with distinct estrogen receptor (ER) statuses (Additional file 1: Fig. S11D).

To further verify the interactions between Hypo-MS4 and the tumor immune microenvironment, we resorted to a dataset with matched single-cell transcriptomic and methylomic profiles from colorectal cancer (CRC) [57]. Our data showed that the fractions of cancer cells with high Hypo-MS4 activity were consistently enriched in lymph node metastases and liver metastases compared with those in primary tumors (Fig. 5B). To leverage other transcriptome datasets of the tumor immune microenvironment and ICI treatments where methylome information is unavailable, we derived a 156-gene expression signature (GES) for Hypo-MS4 by correlating single-cell gene expression levels to Hypo-MS4 activity (AUC = 0.88; Additional file 1: Supplementary Methods; Additional file 1: Fig. S11E) [57]. With single-cell RNA-Seq data from HNSC, we observed the same positive correlation between the fraction of cancer cells with high Hypo-MS4-GES scores and lymph node metastasis [58] (Additional file 1: Fig. S11F). Moreover, in another study of single-cell transcriptome data from colorectal cancer in which tumor-infiltrating lymphocytes were profiled along with tumor cells [59], our data showed that the fraction of cancer cells with a high Hypo-MS4-GES was positively correlated with the fraction of regulatory CD4 + T cells (Pearson’s *R* = 0.46, *P* = 0.028; Fig. 5C) and negatively correlated with the fraction of tumor-infiltrating CD8^+^ T cells (Pearson’s *R* = -0.36, *P* = 0.087) (Additional file 1: Fig. S11G), both of which promote immune evasion of tumor cells [60, 61].

Prompted by these findings, we examined whether Hypo-MS4 was predictive of the response to immune checkpoint inhibitors (ICIs). To this end, we analyzed Hypo-MS4 activity in a dataset of advanced metastatic melanoma patients who received anti-PD1 therapy (*n* = 43), in which DNA methylation was profiled prior to therapy [43]. We found that the activity of Hypo-MS4 was greater in the nonresponders to the PD-1 inhibitor than in the responders (Wilcoxon’s rank sum test, *P* = 0.038) (Fig. 5D, left panel). In another cohort of melanoma patients who received anti-CTLA4 or anti-CTLA4 + anti-PD1 therapy (*n* = 35) [44], Hypo-MS4 was consistently associated with poor responses but was less significant (Fig. 5D, right panel, *P* = 0.36). We then compared Hypo-MS4 activity between ICI responders and nonresponders among patients with melanoma before treatment based on bulk gene expression profiles [45–47]. As a result, Hypo-MS4-GES scores were consistently greater in nonresponders than in responders to ICI treatment in all three cohorts (gastric cancer, Kim et al. 2018, *P* = 0.025; melanoma, Liu et al. 2019, *P* = 0.019; melanoma, Riaz et al.2017, *P* = 0.51; Wilcoxon’s rank sum test) (Fig. 5E). Finally, in a single-cell RNA-seq dataset of melanoma [48], our data consistently showed that the fraction of cancer cells with high Hypo-MS4-GES scores was greater in nonresponders to ICI before treatment (baseline, *P* = 0.156; Wilcoxon rank-sum test, Fig. 5F) and further increased after treatment (posttreatment, *P* = 0.013, Wilcoxon rank-sum test), suggesting that Hypo-MS4-GES is strongly predictive of resistance to ICI therapy.

In summary, Hypo-MS4 is strongly associated with biomarkers of known immune processes in the tumor microenvironment. In particular, these associations are coupled with the consistent predictive power of Hypo-MS4 for poor response to immunotherapy in multiple cancer types.

### Determinants of DNA methylation signatures

Inspired by recent advances in cancer epigenetic reprogramming and our previous findings [39], we believe that revealing the underlying biological processes of methylation signatures is important. Here, we performed an integrated analysis to identify possible deterministic genes of the methylation signatures (Methods).

First, we used mutational status as an instrumental variable (IV) to identify genes whose expression levels influenced the methylation signatures (dependent variable; Methods) [62, 63]. As a result, we identified eleven deterministic gene mutations (six frameshift mutations and five missense mutations; Table 1), which significantly affected the activity of the methylation signatures (FDR < 0.05) (Table 1). Notably, Hypo-MS4 activity is associated with both TP53 and FOXA1 (Table 1). We further investigated whether the deterministic genes bind specifically to the DMPs of certain methylation signatures (Methods). As a result, the FOXA1-binding motifs were significantly enriched in Hypo-MS4-related DMPs in the Panc1 cell line (FDR < 0.05; Table 1) [64]. In addition, there were other genes in which the DNA-binding motifs were significantly enriched in the DMPs related to specific methylation signatures, such as NRF2 (Hypo-MS1), TEAD1 and TEAD3 (Hyper-MS2), RUNX1 (Hypo-MS1, Hypo-MS3), and RUNX2 (Hypo-MS1, Hypo-MS3, Hypo-MS3) (FDR < 0.1, Table 1).

**Table 1:** Instrumental variable regression analysis and motif analysis to determine the deterministic genes of the DNA methylation signatures

	Instrumental variable		Motif	
	Missense	Frame shift	TSS	Gene body
Hyper-MS1			ZNF652(HepG2)	
Hyper-MS2		CDH1	TEAD1(HepG2) MafF(HepG2) LXH9(HCT116) HINFP(K562) TEAD3(HepG2)	Six4(MCF7)
Hyper-MS3			ZNF652(HepG2)	
Hypo-MS1	KEAP1		Jun-AP1(K562) Atf3(GBM) Fra1(BT549) NRF2(HepG2)	Jun-AP1(K562) Fra1(BT549) Atf3(GBM) NF-E2(K562) Bach1(K562) NRF2(HepG2) RUNX2(PCa) EIk1(Hela) ELF5(T47D)
Hypo-MS2	AKAP1 C16orf2	FOXA1	NFIL3(HepG2) Atf1(K562)	Jun-AP1(K562) Fra1(BT549) Atf3(GBM) NF-E2(K562) AR-halfsite(LNCAP)
Hypo-MS3			PSE(K562) RUNX2(PCa) Jun-AP1(K562) Fra1(BT549) Atf3(GBM) NF-E2(K562) RARa(K562)	Jun-AP1(K562) Fra1(BT549) Atf3(GBM) Bach1(K562) NF-E2(K562) c-Myc(LNCAP) Err $\alpha$(HepG2) bHLHE40(HepG2) Max(K562)
Hypo-MS4	**TP53**	TP53 **FOXA1**	COUP-TFII(K562)	**Forkhead (Panc1)** HRE(HepG2)
Hypo-MS5	AKAP9			HNF4a(HepG2)
Hypo-MS6	INHBE	BAP1	Bach1(K562)	
Hypo-MS7		BAP1 BMPR2	AP-2alpha (Hela) Fra1(BT549) RUNX2(PCa)	Atf3(GBM) Fra1(BT549) Jun-AP1(K562)

Our data strongly suggest that the activities of the methylation signatures are determined by the interaction of cancer driver mutations and genes with specific DNA-binding activities.

### Hypo-MS4 surrogates for methylation at FOXA1-binding sites and dependent on TP53 mutation

**Fig. 6:**
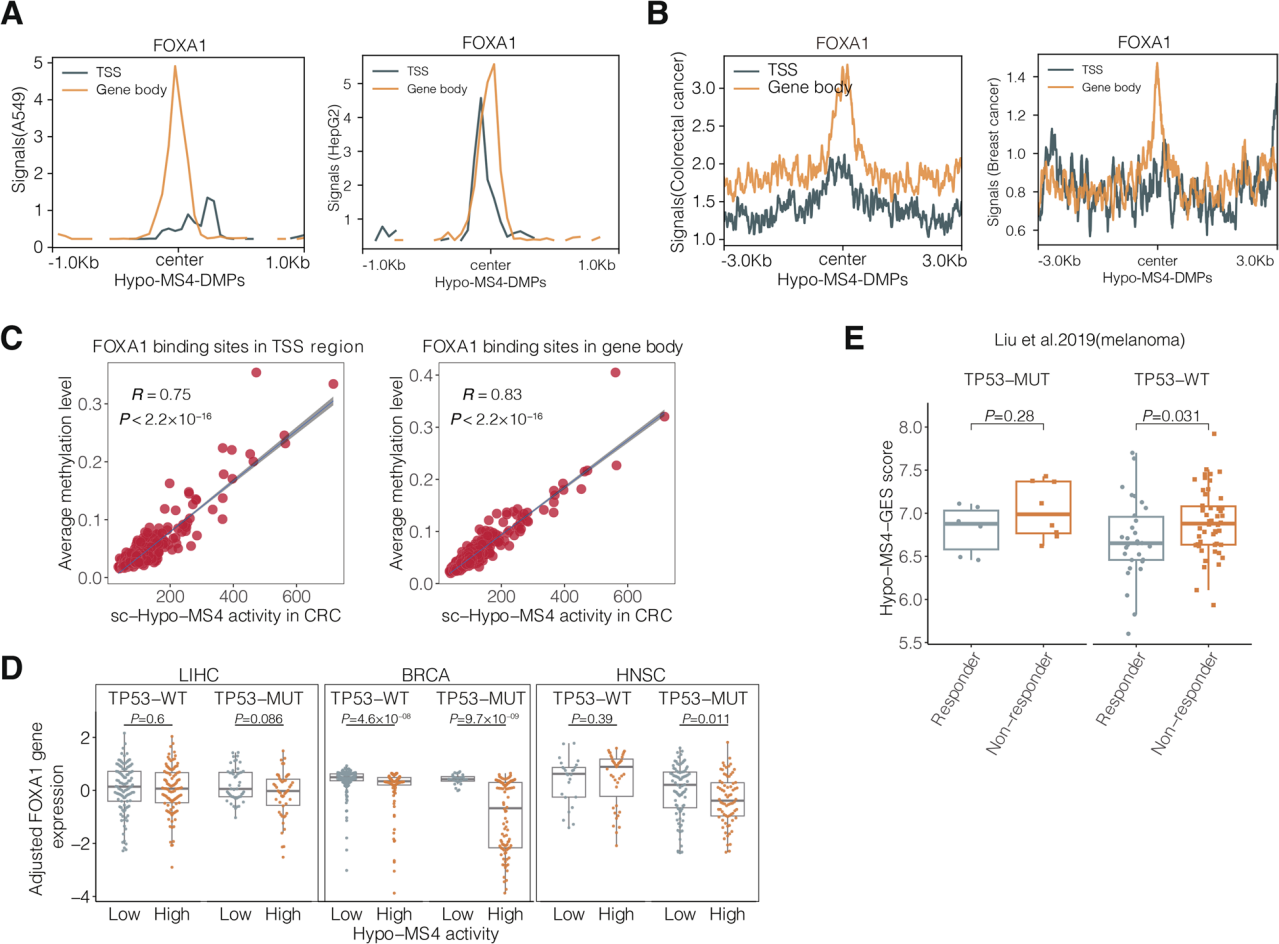
Correlation between deterministic genes and Hypo-MS4 activity. **A** FOXA1 binding landscape of Hypo-MS4-related DMPs in the gene body (*n* = 162) and TSS regions (*n* = 98) in the A549 and HepG2 cell lines. **B** FOXA1 binding landscape of Hypo-MS4-related DMPs in the gene body (*n* = 162) and TSS regions (*n* = 98) in colorectal and breast cancer tissues. **C** Correlation between the mean methylation level of FOXA1-binding sites and Hypo-MS4 activity in single-cell methylome data of colorectal cancer patients (left panel: FOXA1-binding sites in the TSS; right panel: FOXA1-binding sites in the gene body). **D** FOXA1 expression levels in patients with low and high Hypo-MS4 activity (according to the median Hypo-MS4 activity) in TP53-MUT and TP-53 WT tumors from LIHC, BRCA, and HNSC. **E** Hypo-MS4-GES scores increase in TP53-MUT and TP53-WT melanoma patients who are nonresponders to immune therapy

Our data showed that the activity of Hypo-MS4 is strongly influenced by TP53 and FOXA1. We retrieved the FOXA1-binding landscape for the DMPs related to Hypo-MS4 in multiple cancer cell lines (A549, HepG2, T47D and MCF-7) (Fig. 6A and Additional file 1: Fig. S12A, B) and tumor tissues (BRCA and COAD) (Fig. 6B) and showed that the FOXA1-binding affinity selectively peaked at the center of the DMPs. Moreover, the mean methylation levels of FOXA1-binding sites were strongly correlated with the activity of Hypo-MS4 in both TCGA (Additional file 1: Fig. S12C) and single-cell cohorts (Fig. 6C). On the other hand, we did not find any FOXA1-binding affinity at Hypo-MS4-down-DMPs (Hypo-MS4 based on 1-*β*) (Methods, Additional file 1: Fig. S12D).

We further verified the impact of the TP53 mutational status on Hypo-MS4 activity in cancer types in which both Hypo-MS4 and TP53 mutations were present, with a sample size greater than 10. The results revealed that Hypo-MS4 activity increased with increasing TP53 mutation in 3 out of 4 cancer types, namely, LUAD (*P* = 0.81), LIHC (*P* = 0.069), and BRCA (*P* < 2.22 $\times {10}^{-16}$) (Additional file 1, Fig. S12 E, F). In addition, we conducted an analysis of the most commonly recognized hotspot mutations in TP53, namely, R175H, R248Q, R273H, Y220C, and R249S. However, due to the limited number of observations within each TP53 mutant group, we were only able to detect a significant increase in Hypo-MS4 activity within the TP53 (R175H) group in BRCA compared to that in the TP53-WT group (Additional file 1: Fig. S12G).

Next, we sought to evaluate the interactive effects of TP53 and FOXA1 on Hypo-MS4 activity. We observed a negative association between Hypo-MS4 activity and FOXA1 expression in the TP53-MUT group in 3 out of the 4 mentioned cancer types (LIHC: *P* = 0.086; BRCA: *P* = 9.7 $\times {10}^{-9}$; HNSC: *P* = 0.011; Wilcoxon rank-sum test, Fig. 6D and Additional file 1: Fig. S13A). However, in the TP53-WT group, the effect was significant only for BRCA cells, and the effect also interacted with ER status (Fig. 6D, Additional file 1: Fig. S13B). In addition, we performed multilinear regression analysis (Methods) to further validate the interaction effect of FOXA1 and TP53 on Hypo-MS4 activity. The interaction between FOXA1 expression and TP53 mutational status was found to be a significant predictor of decreased Hypo-MS4 activity in 3 out of 4 cancer types (LIHC: coeff =  − 97.68, *P* = 9.4 $\times {10}^{-4}$; BRCA: coeff =  − 54.40, *P* = 0.028; HNSC: coeff =  − 81, *P* = 0.03; and LUAD: coeff = 7.6, *P* = 0.8; Additional file 1: Fig. S13C). Our results suggest that Hypo-MS4 activity in cancer cells is determined by TP53 mutation, FOXA1 expression, and their interaction. FOXA1 can demethylate its binding sites in a POLB-dependent manner [65]. Our observation of TP53 mutant tumors consistently showed that suppression of FOXA1 resulted in reestablishment of methylation at Hypo-MS4-DMPs (FOXA1 binding sites), hence increasing Hypo-MS4 activity.

We further investigated whether the predictive power of Hypo-MS4 is influenced by the TP53 mutational status and FOXA1 expression. In LIHC, KIRC, and KIRP, Hypo-MS4 showed no significant association with OS in patients with TP53-WT or TP53-MUT tumors, suggesting that the effect of Hypo-MS4 on survival is mainly driven by the mutational status of TP53 (Fig. 3A, Additional file 1: Fig. S14A). The same effects were observed within high- and low-FOXA1-expressing tumors (Additional file 1: Fig. S14B). Nevertheless, we observed a consistent increase in Hypo-MS4 activity in melanoma patients who had a poor response to ICIs [46], within the TP53-WT (*P* = 0.031) or TP53-MUT (*P* = 0. 28, Fig. 6E). The effect remained significant after we adjusted for TP53 mutational status and FOXA1 expression in a multivariate logistic regression. (OR_Hypo-MS4_ = 0.74, *P* = 0.02; Additional file 1: Fig. S14C; Methods). Furthermore, neither the mutational status of TP53 nor FOXA1 expression alone predicted an ICI response (Additional file 1: Fig. S14D, E). These findings imply that Hypo-MS4 could serve as an independent predictor of poor ICI response.

## Discussion

Profiling the DNA methylome in cancer populations provides a comprehensive view of epigenetic reprogramming before large parallel ChIP-Seq assays for tumor specimens are available. Distinct patterns of DNA methylation are used to identify subgroups of cancer with functional and clinical implications and are key to understanding tumorigenesis and somatic evolution. Nevertheless, the current definition of methylation signatures is based on highly focal methylation patterns in CpG islands (CIMP) or methylation patterns in tumors of specific tissue origins. Our analysis demonstrated the existence of conserved signatures of DNA methylation at the pan-cancer level and revealed distinct cancer processes, such as response to cancer and treatment.

Many previous studies have reported cancer genes with diverse functional impacts based on aberrant DNA methylation status in situ [53]. In the present study, we considered the landscape of the cancer methylome to be a result of multiple endogenous and exogenous processes. As DNA methylation is the result of many heterogeneous processes that occur in both normal and cancer cells, our study focused on a set of highly conserved differentially methylated DNA positions in cancer or on DMP. To define DMP, we performed a large-scale permutation test with stringent control for unspecific variation based on paired methylome data and adjusted for cancer types. Thus, the methylation signatures were retrieved on the basis of conserved DMP representing systematic variation in the cancer methylome and surrogate specifically for cancer-related biological processes.

Our study is also inspired by many previous pan-cancer studies in coping with the technical challenges in solving the complex dynamics of DNA methylation. To minimize the effects of contaminating normal tissue, we used the difference in beta values between matched tumor and normal specimens as a measure of DNA methylation. We chose NMF, which enforces nonnegativity for both the signature scores and the coefficients, for the analysis. In addition, NMF imposes no orthogonality or independence constraints, therefore permitting partially correlated methylation signatures, which also improves the interpretability of the results. Notably, according to the results of NMF, highly active hypomethylation signatures are interpreted as an increased proportion of methylated sites that tend to be demethylated in cancer cells. This observation is consistent with previous observations [66] that even extensively hypomethylated positions can also be hypermethylated. Our findings thus provide a more comprehensive view of the variation in the cancer-associated methylome and thereby suggest new determinants of tumorigenesis and clonal evolution.

In addition, although NMF does not incorporate prior information, the resulting methylation signatures demonstrated strong tissue specificity. Despite the fact that the signatures are active only in certain cancer types, some methylation signatures are present in multiple cancer types. With respect to the pan-cancer methylation signatures, we demonstrated relatively consistent molecular and clinical characteristics, suggesting that conserved pan-cancer biological processes underlie these signatures. Moreover, the methylation signatures reflect the variability of the methylation levels at the DMPs in cancer cells. However, our data suggest that certain Hypo-DMPs in Hypo-MS4 are remethylated in cancer. Such remethylation of Hypo-DMPs in cancer tissues does not change the characteristic low methylation levels of these DMPs compared with those in normal tissues. In addition, the variations in methylation in DMPs corresponding to certain methylation signatures are highly conserved, despite the activity, methylation, or demethylation used to define it.

Our findings showed that the cancer methylome interacts with important biological processes to determine the molecular and clinical phenotypes of cancer. These interactive effects are revealed by the activity of unique methylation signatures, which we further traced back to the cancer driver genes. To this end, we not only reaffirmed previous case observations but also suggested specific functional regulators at a systematic level. As a major finding, we demonstrated that Hypo-MS4 mediates immune responses to neoantigens in cancer, which is dependent on TP53 mutation and FOXA1 activity. TP53 mutations are well known to impact tumor immune evasion and promote cancer progression [65]. FOXA1 is a pioneer of epigenetic reprogramming in both stem cells and cancer cells and is involved in DNA repair complex formation and DNA demethylation [67, 68]. Although both genes are known cancer drivers, the cooperative role of these two genes in cancer has largely not been fully elucidated until recently [69]. Nevertheless, the association between Hypo-MS4 and neoantigen load as well as the suppression of immune responses in the TIME further strengthened the relationship between FOXA1 activity and cancer immunity. Overall, we hypothesize that Hypo-MS4 surrogates FOXA1 activity to modulate the cancer methylome, hence interfering with neoantigen presentation and subsequent immune responses in the microenvironment.

Our results also lead to several strong clinical implications. First, we showed that Hypo-MS4 was associated with poor clinical outcomes. This effect was largely driven by TP53 mutation, which was dependent on Hypo-MS4. We subsequently showed that Hypo-MS4 is associated with distant metastases and resistance to immune therapy, which is independent of TP53 and FOXA1 and is associated with the impacts of Hypo-MS4 on immune responses. To this end, Hypo-MS4 not only provides new evidence for the clinical impact of FOXA1 driver mutations but also serves as a more sensitive, quantifiable indicator of the tumor immune status with diagnostic and therapeutic potential.

In particular, we demonstrated how cancer-related transcription factors, such as FOXA1, act as modulators of DNA methylation at specific loci and thereby reprogram the cancer epigenome to alter relevant biological functions. The consequential changes in molecular and clinical phenotypes further inform the prediction and treatment of cancers. Nevertheless, similar mechanisms may exist for other DNA-binding proteins and epigenetic regulatory enzymes in cancer. With more advanced sequencing technology, we will be able to identify other modulators of epigenetic reprogramming in cancers.

However, for the other methylation signatures, the biological backgrounds are still unclear. Many of these methylation signatures can be observed only in cancer tissues and not in cell lines. These signatures can be driven by either exogeneous factors such as environmental exposure and therapies or by other endogenous effects such as genetic variants [30, 68, 70]. In either case, the availability of high-resolution DNA methylation profiles will increase the interpretability of these methylation signatures [71, 72]. Moreover, the significance of molecular and clinical evidence in different cancer types is limited by sample size and uncontrolled confounders. However, further analyses in large, well-controlled cohorts are needed to verify the findings of the present study. Nevertheless, methylation signatures also enable the accurate identification of single DMPs associated with specific biological processes in cancer, such as neoantigens or cis-regulatory elements of cancer genes.

Finally, the activities of the methylation signatures are, by definition, average levels of subsets of DMPs of different sizes. Therefore, different methylation signatures should be compared with caution. Although the scores of methylation signatures reflect the actual proportion of methylation at the DMPs, each signature corresponds to biological processes affecting a unique subset of DMPs. Because there is currently no standardization for methylation signatures, conclusions based on direct comparisons of different methylation signatures need to take into account the different sizes of DMPs affected.

In summary, we have described the derivation of a set of conserved pan-cancer DNA methylation signatures and studied the underlying etiology and molecular mechanisms. Our data demonstrate how cancer methylation reprogramming interacts with somatic mutations, the microenvironment, and immune responses and impacts the molecular and clinical phenotypes of cancer. We also report that FOXA1 is a modulator of a specific methylation signature. Our findings provide new insights for the search for novel diagnostic and therapeutic targets for cancer treatment.

## Conclusions

Our data revealed conserved biological processes that shape the landscape of the cancer methylome and are represented by signatures of altered methylation at specific loci. In addition, the methylation signatures in cancer are tightly coupled with epigenetic reprogramming and immune responses in the microenvironment. In particular, known cancer-related transcription factors such as FOXA1 act as modulators of DNA methylation at binding sites. Finally, the activities of the methylation signatures can influence specific molecular and clinical characteristics of cancer.

### Supplementary Information

**Additional file 1: Fig S1.** Distribution of DMPs’ median-∆β values between tumor and normal tissues at the pan-cancer level. **Fig S2.** Top 10 significant Hyper-DMPs across 9 cancer types. **Fig S3.** Top 10 significant Hypo-DMPs across 9 cancer types. **Fig S4.** Identification of conserved differentially methylated probes at the pan-cancer level. **Fig S5.** NMF identifies three hypermethylation signatures and seven hypomethylation signatures. **Fig S6.** Comparison of Hypo-MSs using β or 1-β values as input. **Fig S7.** Characterization of DNA methylation signatures. **Fig S8.** Methylation signature activities’ association with age. **Fig S9.** Analysis of the correlations between overall survival, cancer stages and methylation signature activities. **Fig S10.** The relationship between methylation signature activities and tumor immune microenvironment in cancers. **Fig S11.** The relationship between Tumor mutation burden, neoantigen load, tumor progression and Hypo-MS4 activity. **Fig S12.** Analysis of the correlations between deterministic genes and Hypo-MS4 activity. **Fig S13.** Analysis of the intersection of deterministic genes and Hypo-MS4 activity. **Fig S14.** Analysis of overall survival and ICI response of Hypo-MS4 with deterministic genes status.

**Additional file 2: Table S1.** Sample information. **Table S2.** Variation of DNA methylation signatures attributed to tissue origins. **Table S3.** Determinants of methylation signatures.

## Data Availability

The lymphocyte activities of the TCGA samples were obtained from https://www.cell.com/cms/10.1016/j.cell.2014.12.033/attachment/fdbc71ba-1786-469f-9a7b-6c05e2ab76c4/mmc1.xlsx [55]. Immune cell fractions from TCGA samples were obtained from https://ars.els-cdn.com/content/image/1-s2.0-S1074761318301213-mmc2.xlsx [56]. The neoantigen loads of nine cancer types in the TCGA cohort are publicly available from http://biopharm.zju.edu.cn/tsnadb [73]. The matched single-cell transcriptomic and methylomic profiles from colorectal cancer (CRC) patients were obtained from the GEO repository (GSE97693; https://www.ncbi.nlm.nih.gov/geo/query/acc.cgi?acc=GSE97693) [57]. Single-cell RNA-seq data of HNSC were downloaded from the GEO (https://www.ncbi.nlm.nih.gov/geo/query/acc.cgi?acc=GSE103322) [58]. The single-cell transcriptome of colorectal cancer was downloaded from the GEO (https://www.ncbi.nlm.nih.gov/geo/query/acc.cgi?acc=GSE132465) [59]. The clinical and Illumina EPIC DNA methylation array datasets of melanoma patients with immune therapy responses were obtained from the GEO with accession numbers GSE181781 (https://www.ncbi.nlm.nih.gov/geo/query/acc.cgi?acc=GSE181781) [43] and GSE175699 (https://0-www-ncbi-nlm-nih-gov.brum.beds.ac.uk/geo/query/acc.cgi?acc=GSE175699) [44], respectively. The bulk RNA-seq data of gastric cancer patients who underwent immune therapy are available from the European Nucleotide Archive under accession number PRJEB25780 (https://www.ebi.ac.uk/ena/browser/view/PRJEB25780) [45]. The bulk RNA-seq datasets of melanoma patients with immune therapy responses were obtained from dbGaP (accession number: phs000452.v3.p1; https://www.ncbi.nlm.nih.gov/projects/gap/cgi-bin/study.cgi?study_id=phs000452.v3.p1) [46] and from the GEO (accession number: GSE91061; https://www.ncbi.nlm.nih.gov/geo/query/acc.cgi?acc=GSE91061) [47]. The single-cell RNA-seq data of melanoma were obtained from the GEO under the accession number GSE120575 (https://www.ncbi.nlm.nih.gov/geo/query/acc.cgi?acc=GSE120575) [48]. All the codes and intermediate data of the analyses are available at Github (https://github.com/xmbd/Pan_Meth_Sig) [74].
