# Supplementary material for: Conserved methylation signatures associate with the tumor immune microenvironment and immunotherapy response
Source: Genome Med. 2024 Apr 2;16:47. doi: 10.1186/s13073-024-01318-3 (PMC10985907; doi:10.1186/s13073-024-01318-3)
Supplement: Supplementary file 1 — Additional file 1: Fig S1. Distribution of DMPs’ median-∆β values between tumor and normal tissues at the pan-cancer level. Fig S2. Top 10 significant Hyper-DMPs across 9 cancer types. Fig S3. Top 10 significant Hypo-DMPs across 9 cancer types. Fig S4. Identification of conserved differentially methylated probes at the pan-cancer level. Fig S5. NMF identifies three hypermethylation signatures and seven hypomethylation signatures. Fig S6. Comparison of Hypo-MSs using β or 1-β values as input. Fig S7. Characterization of DNA methylation signatures. Fig S8. Methylation signature activities’ association with age. Fig S9. Analysis of the correlations between overall survival, cancer stages and methylation signature activities. Fig S10. The relationship between methylation signature activities and tumor immune microenvironment in cancers. Fig S11. The relationship between Tumor mutation burden, neoantigen load, tumor progression and Hypo-MS4 activity. Fig S12. Analysis of the correlations between deterministic genes and Hypo-MS4 activity. Fig S13. Analysis of the intersection of deterministic genes and Hypo-MS4 activity. Fig S14. Analysis of overall survival and ICI response of Hypo-MS4 with deterministic genes status. [file 13073_2024_1318_MOESM1_ESM.docx]

**Supplementary materials**

## Conserved methylation signatures associate with the tumor immune microenvironment and immunotherapy response

Qingqing Qin1,2,3†, Ying Zhou1,2,3†, Jintao Guo1,2,3, Qinwei Chen1,2, Weiwei Tang4,5, Yuchen Li1,2,3, Jun You^6^ and Qiyuan Li1,2,3*

1 Department of Hematology, The First Affiliated Hospital of Xiamen University and Institute of Hematology, School of Medicine, Xiamen University, Xiamen, 361102, China. 2 National Institute for Data Science in Health and Medicine, School of Medicine, Xiamen University, Xiamen, 361102, China. 3 Department of Pediatrics, The First Affiliated Hospital of Xiamen University, Xiamen, 361102, China. 4 Department of Medical Oncology, The First Affiliated Hospital of Xiamen University and Institute of Hematology, School of Medicine, Xiamen University, Xiamen, 361102, China. 5 Xiamen Key Laboratory of Antitumor Drug Transformation Research, The First Affiliated Hospital of Xiamen University, School of Medicine, Xiamen University, The School of Clinical Medicine of Fujian, Medical University, Xiamen, 361102, China. 6 Department of Gastrointestinal Oncology Surgery, Cancer Center, The First Affiliated Hospital of Xiamen University, Xiamen, 361102, China

*Correspondence to Qiyuan Li, National Institute for Data Science in Health and Medicine, School of Medicine, Xiamen University, Xiamen, 361102, China, Email: qiyuan.li@xmu.edu.cn

† These authors contributed equally to this work.

**Supplementary methods**

**Fig S1 - S14**

**Supplementary methods**

### Tumor sample classification using non-negative matrix factorization (NMF) in TCGA

We clustered the tumor samples into "signature-positive" and "signature-negative" groups using the "predict" function and the default setting in NMF. The sample assignment depended on the relative values in each column of Signature Matrix “S”.

### Conserved DNA methylation signature in cancer cell lines

We derived DNA methylation signatures for hyper- and hypo-DMPs in 241 cancer cell lines using the aforementioned method. Additionally, based on the same rank selection in tissue, we chose the number of signatures, k = 2 for both the hypermethylation and hypomethylation signatures in cancer cell lines.

Then, to evaluate the methylation signatures’ conservation between TCGA and cell lines, we compared the similarity between the cancer cell line and TCGA sample derived E (weight) matrix using spearman correlation coefficients.

## Variation of methylation signature determined by cancer types

To evaluate the fraction of total variation of each signature contributed by the cancer types. We calculate the variation between groups (cancer types, $V_{\mathrm{between}}$) and within groups (samples, $V_{\mathrm{within}}$) for each methylation signature as following, respectively.

$V_{between}=\sum_{i}^{K} n_{i}(\mu_{i}-\mu)^{2} (1)$

$\mu_{i}$ represents the sample mean of the $i_{th}$ group, $\mu$ was the overall population mean of all samples.

$$V_{within}=\sum_{i=1}^{K} \sum_{j=1}^{n_{i}} (x_{ij}-\mu_{i})^{2} \left( 2 \right)$$

$x_{ij}$ represents the $j_{th}$ sample value in the $i_{th}$ group, $\mu_{i}$ is the sample’s mean of the $i_{th}$ group, $n_{i}$ is the sample size of the $i_{th}$ group.

Then, we calculate the variations ratios of the cancer types ($V_{\mathrm{cancertypes}}$):

$$V_{\mathrm{cancertypes}}=\frac{V_{\mathrm{between}}}{V_{\mathrm{between}}+V_{\mathrm{within}}} \left( 3 \right)$$

### Genes associated with methylation signatures

We performed a linear regression analysis for gene expression levels and methylation signatures, adjusting for cancer type (Fig. 4F). We selected significant signature-related genes with adjusted p values less than 0.1 (method = Benjamini & Hochberg).

$${mRNA}_{ij}=\beta_{0} +{\beta_{1}\times MS}_{ki}+{\beta_{2}\times C}_{i}+\varepsilon_{ijk} \left( 4 \right)$$

Here, $\varepsilon_{\mathrm{ijk}}\sim N \left( 0,\sigma^{2} \right)$ is a Gaussian error term; ${mRNA}_{ij}$ is the $j_{th}$gene expression of the $i_{th}$ sample; ${MS}_{ki}$ is the $k_{th}$ methylation signature of the $i_{th}$ individual; $C_{i}$ is the cancer type of the $i_{th}$ sample; $\beta_{0}$ is the intercept; $\beta_{1}$ is the regression coefficient for methylation signature; and $\beta_{2}$ is the regression coefficient for the cancer type.

### Reproducing methylation signatures in single-cell WGBS datasets

Single-cell WGBS of colorectal cancer and matched adjacent normal cells were obtained from the GEO database (accession number GSE97693). In the single-cell methylome, the methylation sites are measured genome-wide, from several million to hundreds of millions, hence the signals are relatively sparse. The TCGA 450K data only measures the methylation of selected CpG islands. Therefore, the methylation positions in the single-cell methylome are not directly mapped to those in the 450K data. Currently, there is no direct mapping between single-cell whole-genome methylation data and 450K methylation microarray data.

In this study, we used the R package "methylKit" (Methods) to assign single-cell DNA methylation positions into consecutively segmented methylation levels using a Gaussian mixture model. The methylation value of each segment is represented by the average of all methylation positions. Each segment contains up to 1000 of methylation positions with similar methylation statuses, covering up to 3,000 base pairs.

We intersected all the methylation segments from single-cell methylome data with the probes of the 450K data. The methylation level of a corresponding probe is then determined by the segment which covers the probe. If multiple probes all match the same segment, the probes all have the same methylation levels as the segment. Thus, we ensured no missing values in the resultant probe-level methylation values from single-cell whole genome methylation data. The average methylation value of all loci in a given segment was used as the methylation level ($\beta$ value) of the corresponding position. We then mapped these methylation segments to the corresponding Infinium 450K methylation probe using “BEDTools”. Thus, we obtained metrices, $V_{c}$, of methylation levels for $n$single cells and of the same dimension $p$ as the β-value matrices of hyper- and hypo-DMPs.

We then calculated each cell’s signature scores using predefined loading matrices from the NMF in the TCGA samples ($E_{1}$) and in cancer cell lines ($E_{2}$).

$$E_{c}=\frac{E_{1}+E_{2}}{2} \left( 5 \right)$$

The score matrix of a k methylation signatures was given as:

$$H_{c}= E_{c}^{-1}\times V_{c} \left( 6 \right)$$

Finally, to determine the binary status of a given methylation signature in a single cell, we divided cell activity greater than the median value of Hypo-MS4 as high and low Hypo-MS4 cells.

### Defining gene expression of signature

As described in previous studies [1-3], methylation data were unavailable. Therefore, we derived a methylation signature gene expression score (MS-GES) to represent the methylation data. Notably, we utilized sc-RNA seq and sc-WGBS data from Bian et al. [4] Finally, we used a multilinear model to calculate the association between the mRNA expression levels and methylation signature activity in the same cell.

$$\begin{aligned} {mRNA}_{ij}=\beta_{0}+\beta_{1}\times{MS}_{ik}+\beta_{2}\times{TO}_{ik}+\varepsilon_{ijk}\#\left( 7 \right) \end{aligned}$$

$\mathrm{mRNA}_{\mathrm{ij}}$ is the $j_{th}$ gene expression of the $i_{th}$ cell, ${MS}_{ki}$ is the $k_{th}$ methylation signature of the $i_{th}$ cell; $\beta_{0}$ is the intercept, $\beta_{1}$is the regression coefficient for methylation signature; and $\beta_{2}$ is the regression coefficient for the tissue of origin. We selected significant genes with $\beta_{1}$>0, FDR<0.1. Then, we narrowed down the gene list by overlapping the above significant genes with 5,000 highly variable genes (HVGs, R package, seurat) in a single-cell analysis. Finally, we chose 156 genes with the best ROC curve (R package pROC) as representative signature genes of Hypo-MS4. Additionally, we calculated the median of these signature genes’ expression levels and called them methylation-associated gene expression scores (MS-GES scores). Next, we used MS-GESs as substitutes for methylation signature activity to investigate the relationship between methylation signatures and cancer immunotherapy in studies without methylation data. Finally, we compared the MS4-GES scores between ICI responders and non-responders using the Wilcoxon test in bulk RNA-seq and sc-RNA seq.

For sc-RNA seq data, we classified every single cell into cancer cells with high Hypo-MS4-GES score and cancer cells with low Hypo-MS4-GES score according to the median of Hypo-MS4-GES scores. We then calculated the fractions of cancer cells with high Hypo-MS4-GES scores and low Hypo-MS4-GES scores between ICI responders and non-responders.

### Correlation between Hypo-MS4 activity and the methylation level of FOXA1 binding sites

To further validate the correlation between the methylation levels of the FOXA1 binding sites and the Hypo-MS4 activity, we calculated the average methylation levels of FOXA1 binding sites (TSS and Gene body regions) using a single cell methylome data of colorectal cancer[4] and cancer tissues (LUAD, LIHC, BRCA and HNSC) from TGCA. And we used Spearman correlations to assess the relationship between Hypo-MS4 activity and the methylation level of FOXA1 binding sites.

### Deterministic genes influence the predictive power of Hypo-MS4

To verify the impacts of TP53 and FOXA1 on the predictive power of Hypo-MS4. We used a multivariate linear model to evaluate the relation between ICI response and TP53 mutation, FOXA1 gene expression and Hypo-MS4-GES score in a melanoma dataset[1]:

$${ICI}_{i}=\beta_{0} +{\beta_{1}\times MS4}_{i}+{\beta_{2}\times FOXA1}_{i}+{\beta_{3}\times TP53}_{i}+\varepsilon_{i} \left( 8 \right)$$

Here, $\varepsilon_{i}\sim N\left( 0,\sigma^{2} \right)$ is a Gaussian error term; ${ICI}_{i}$ (0 represents non-responder, 1 represents responder) is the response of the $i_{th}$ patient; ${MS4}_{i}$ is the Hypo-MS4-GES score of the $i_{th}$ patient; ${FOXA1}_{i}$ is the FOXA1 gene expression of the $i_{th}$ patient; ${TP53}_{i}$ is the TP53 mutation status of the $i_{th}$ patient; $\beta_{0}$ is the intercept; $\beta_{1}$the regression coefficient for Hypo-MS4-GES; and $\beta_{2}$ is the regression coefficient for FOXA1 gene expression; and $\beta_{3}$ is the regression coefficient for TP53 mutation.

References

1. Liu D, Schilling B, Liu D, Sucker A, Livingstone E, Jerby-Arnon L, et al. Integrative molecular and clinical modeling of clinical outcomes to PD1 blockade in patients with metastatic melanoma. Nat Med. 2019, 25**:**1916-1927.

2. Kim ST, Cristescu R, Bass AJ, Kim KM, Odegaard JI, Kim K, et al. Comprehensive molecular characterization of clinical responses to PD-1 inhibition in metastatic gastric cancer. Nat Med. 2018, 24**:**1449-1458.

3. Riaz N, Havel JJ, Makarov V, Desrichard A, Urba WJ, Sims JS, et al. Tumor and Microenvironment Evolution during Immunotherapy with Nivolumab. Cell. 2017, 171**:**934-949 e916.

4. Bian S, Hou Y, Zhou X, Li X, Yong J, Wang Y, et al. Single-cell multiomics sequencing and analyses of human colorectal cancer. Science. 2018, 362**:**1060-1063.

**List of Supplementary Figures**

**Fig S1. Distribution of DMPs’ median-∆β values between tumor and normal tissues at the pan-cancer level.**

**Fig S2.** **Top 10 significant Hyper-DMPs across 9 cancer types.**

**Fig S3. Top 10 significant Hypo-DMPs across 9 cancer types.**

**Fig S4.** **Identification of conserved differentially methylated probes at the pan-cancer level.**

**Fig S5. NMF identifies three hypermethylation signatures and seven hypomethylation signatures.**

**Fig S6. Comparison of Hypo-MSs using β or 1-β values as input.**

**Fig S7. Characterization of DNA methylation signatures.**

**Fig S8. Methylation signature activities' association with age.**

**Fig S9. Analysis of the correlations between overall survival, cancer stages and methylation signature activities.**

**Fig S10. The relationship between methylation signature activities and tumor immune microenvironment in cancers**.

**Fig S11. The relationship between Tumor mutation burden, neoantigen load, tumor progression and Hypo-MS4 activity.**

**Fig S12. Analysis of the correlations between deterministic genes and Hypo-MS4 activity.**

**Fig S13. Analysis of the intersection of deterministic genes and Hypo-MS4 activity.**

**Fig S14**. **Analysis of overall survival and ICI response of Hypo-MS4 with deterministic genes status.**

**Fig S1**


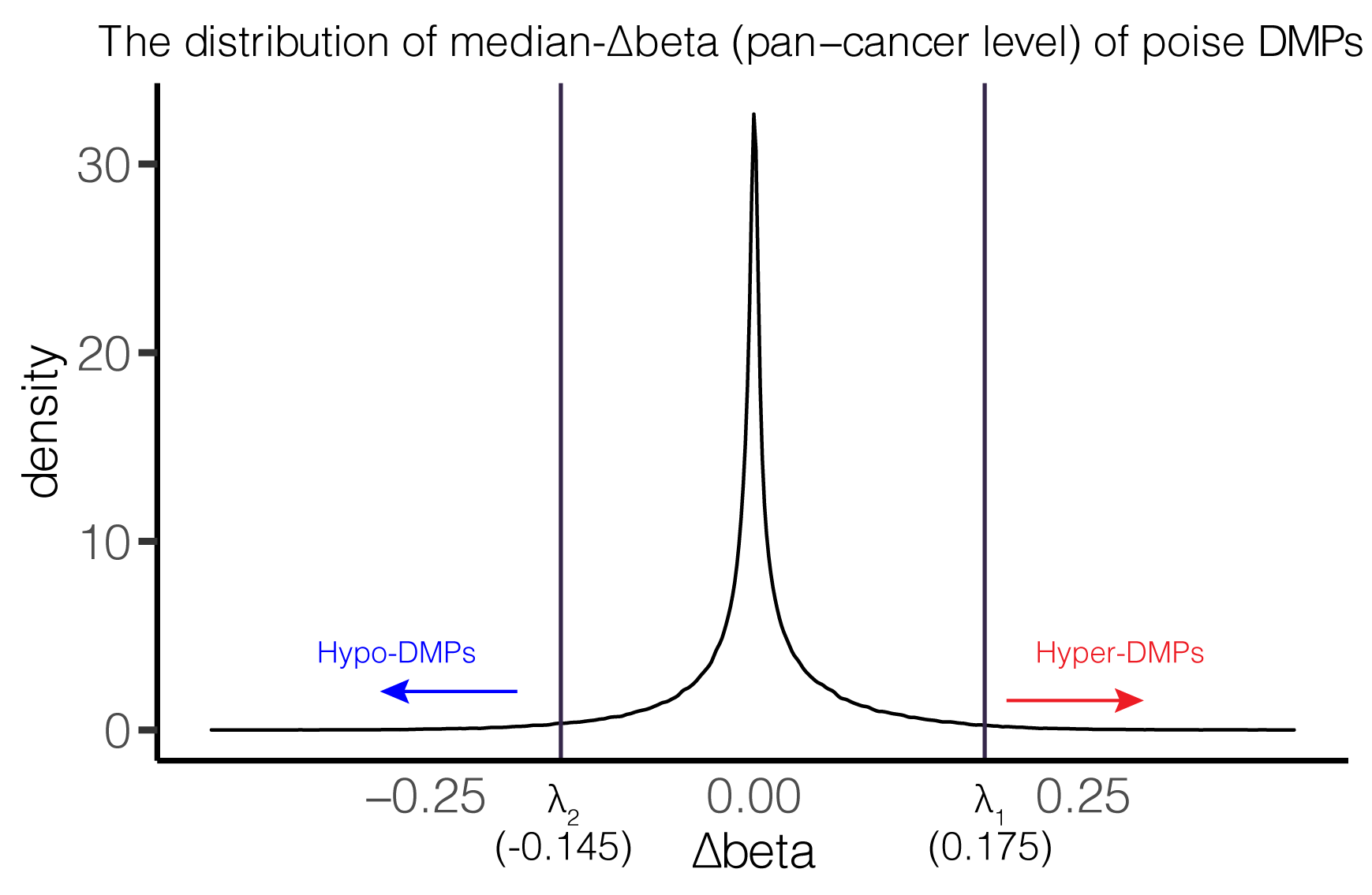


**Fig S1.** **Distribution of median-**$\boldsymbol{\Delta\beta}$ **of poised DMPs.** We defined poised Hyper-DMPs with median-$\Delta\beta$ values greater than λ_1_=0.175 as Hyper-DMPs and poised Hypo-DMPs of which median-$\Delta\beta$ less than λ_2_=-0.147 as Hypo-DMPs.

**Fig S2**

**
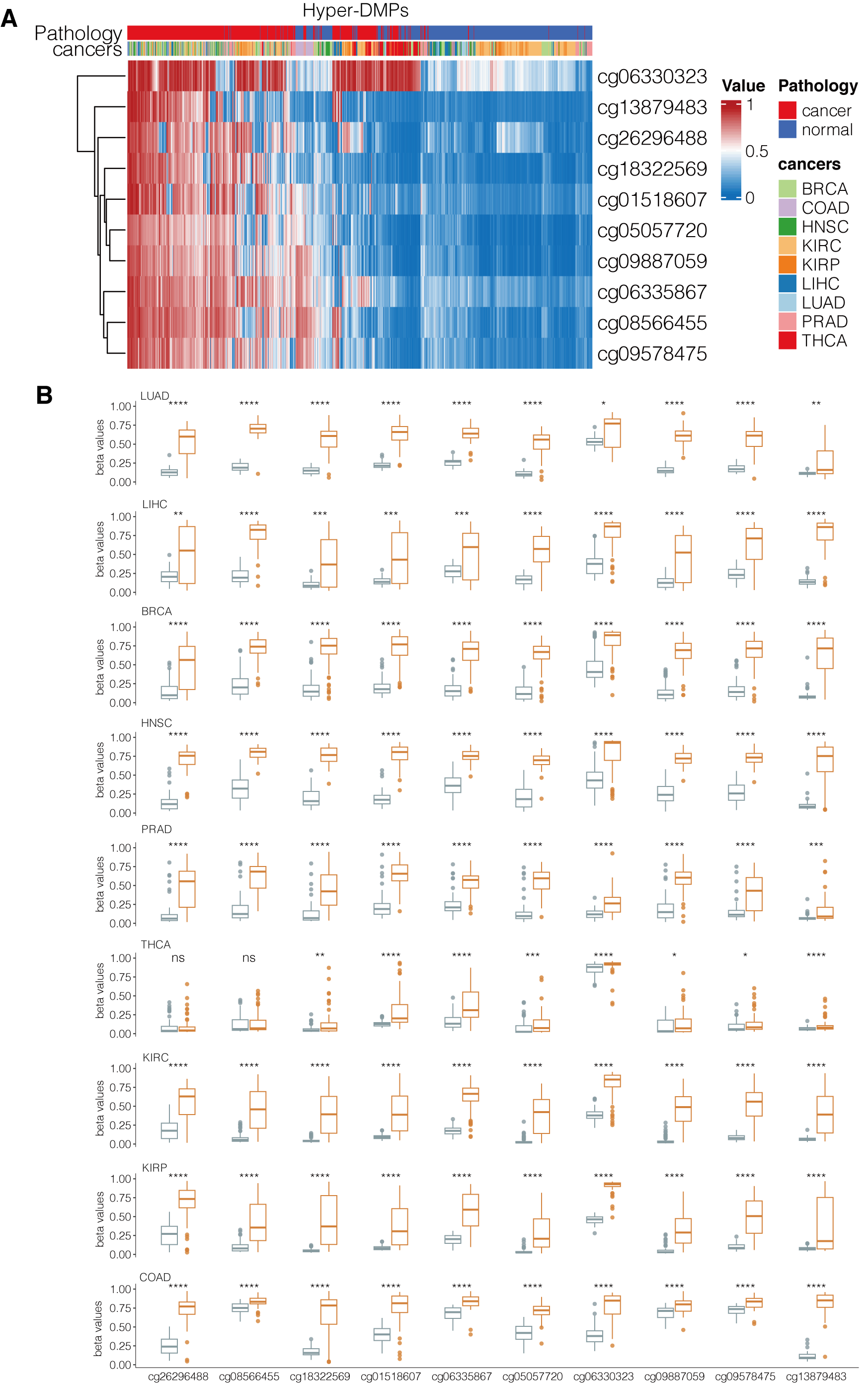
**

**Fig S2. Top 10 significant Hyper-DMPs across 9 cancer types. (A)** The heatmap of top 10 most significant Hyper-DMPs' beta values between tumor tissues and normal tissues in 9 cancer types. **(B)** Boxplots of top 10 Hyper-DMPs between tumor and normal tissue in 9 cancer types. The yellow boxes represent tumor tissues, and the gray boxes represent normal tissues. (Wilcoxon’s rank sum test, ****: P<=0.0001, ***: P<=0.001, **: P<=0.01, *: P<0.05).

**Fig S3**

**
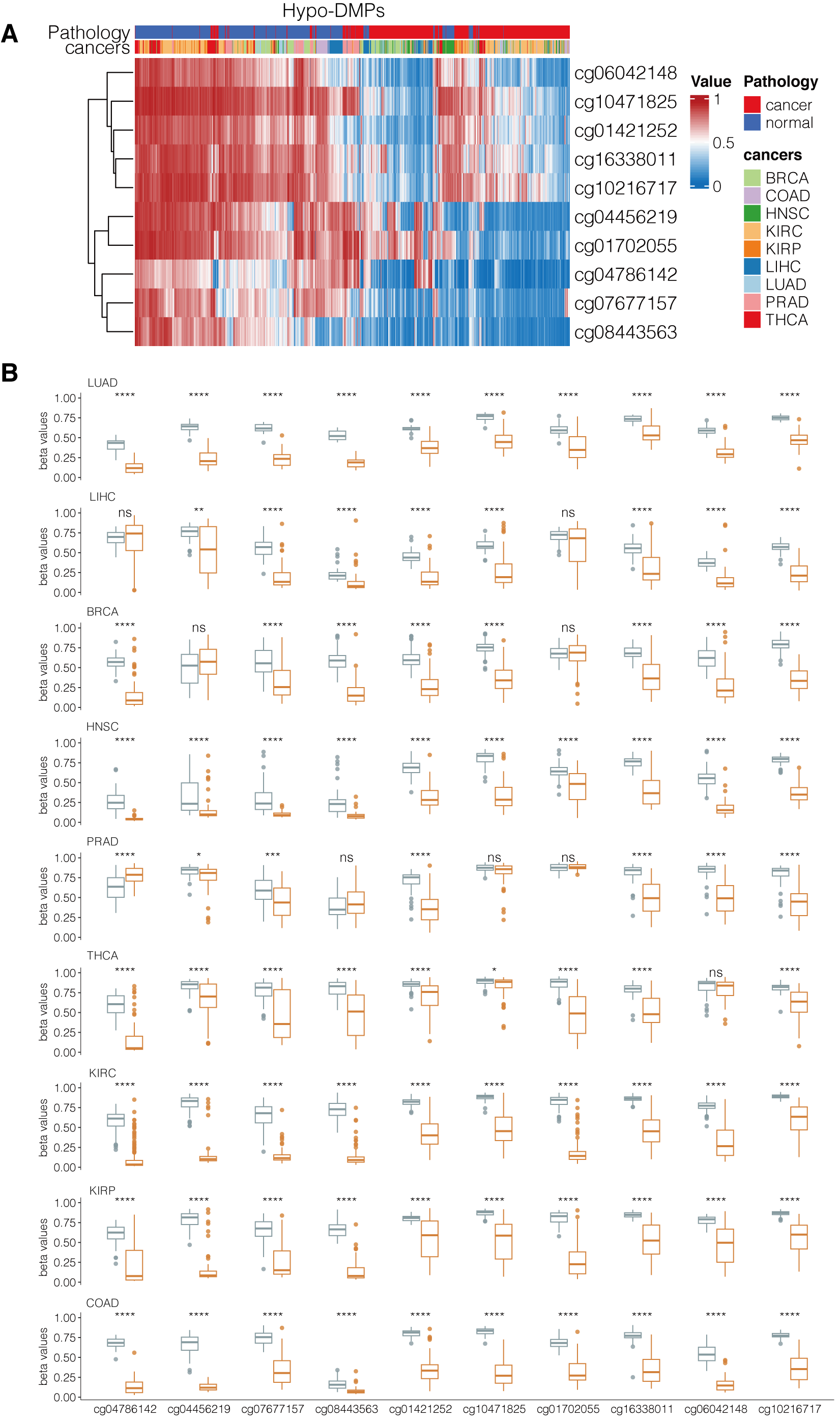
**

**Fig S3. Top 10 significant Hypo-DMPs across 9 cancer types.**

**(A)** The heatmap of top 10 most significant Hypo-DMPs' beta values between tumor tissues and normal tissues in 9 cancer types. **(B)** Boxplots of top 10 most significant Hypo-DMPs between tumor and normal tissues in 9 cancer types. The yellow boxes represent tumor tissues, and the gray boxes represent normal tissues. (Wilcoxon’s rank sum test, ****: P<=0.0001, ***: P<=0.001, **: P<=0.01, *: P<0.05).

**Fig S4**


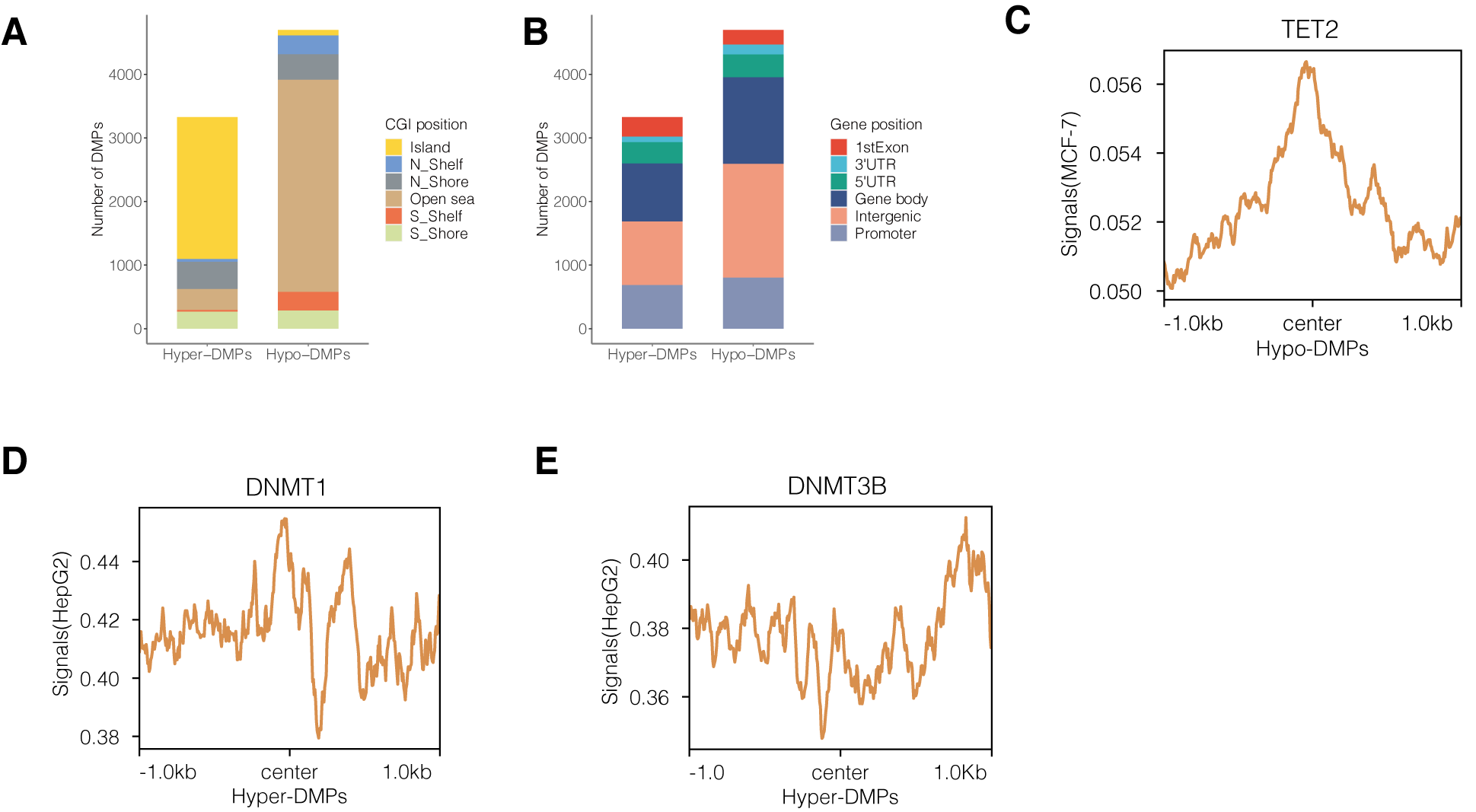


**Fig S4.** **Identification of conserved differentially methylated probes at the pan-cancer level.**

**(A)** Genomic location of Hyper-DMPs and Hypo-DMPs relative to the CpG island. **(B)** Genomic location of Hyper-DMPs and Hypo-DMPs relative to the genes. The y-axis represents the number of DMPs. The length of different colored bars represents the proportion of DMPs distribution. **(C)** TET2 protein binding landscape of 1kb from either side of the center of Hypo-DMPs (N=4,395). **(D-E)** Distribution of DNMT1 and DNMT3B protein binding landscape of 1kb from either side of the center of Hypo-DMPs.

**Fig S5**

**
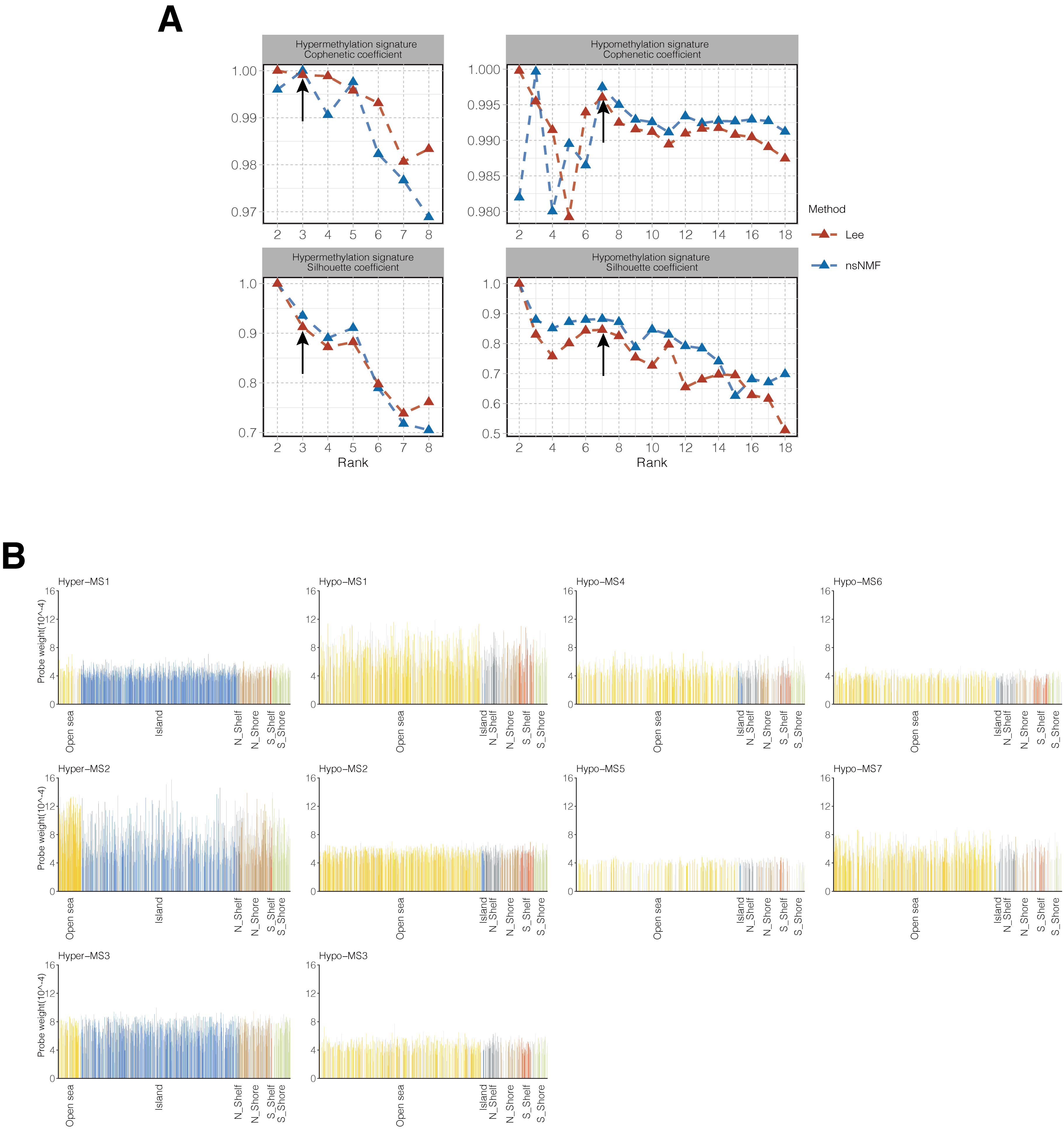
**

**Fig S5.** **NMF identifies three hypermethylation signatures and seven hypomethylation signatures. (A)** Cophenetic and silhouette coefficients for each k in methylation signature extraction using NMF. Different colors represent different methods. The black arrows denote our preferred clusters. **(B)** Decomposition plots of 10 pan-cancer DNA methylation signatures (Hyer-MS1-3，Hypo-MS1-7). Different colors indicate the DMPs’ location in the CGI position.

**Fig S6**

**
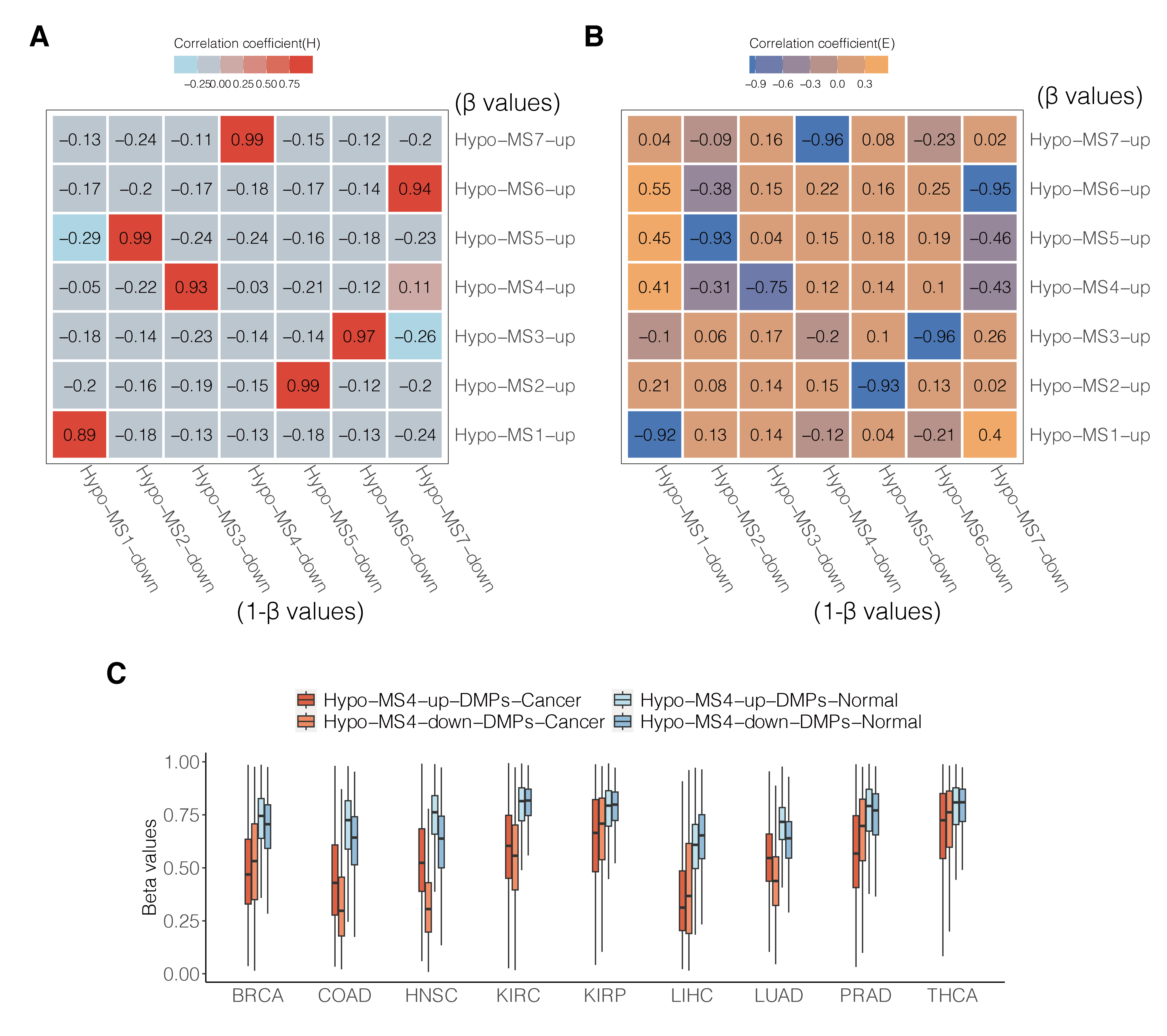
**

**Fig S6. Comparison of Hypo-MSs using β or 1-β values as input. (A)** Correlation coefficients of signature score matrices (Hs) derived from β values and 1-β values of the Hypo-DMPs, respectively. (**B**) Correlation coefficients of weight matrices (Es) corresponding to β values and 1-β values of the Hypo-DMPs, respectively. **(C)** The β values of Hypo-MS4-up-DMPs and Hypo-MS4-down-DMPs in both cancer tissues and normal tissues, respectively.

**Fig S7**


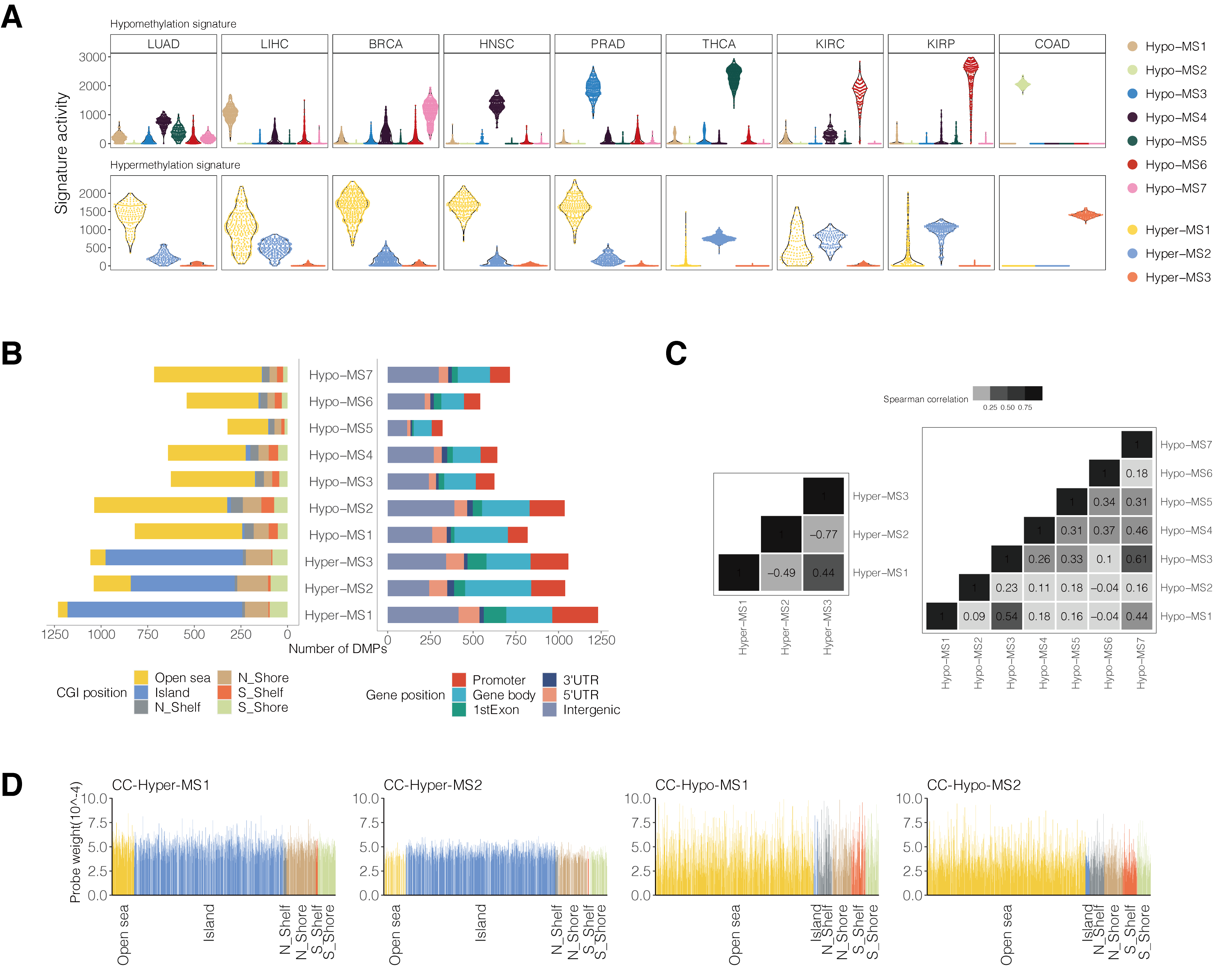


**Fig S7.** **Characterization of DNA methylation signatures.** **(A)** DNA methylation signature activity in each cancer type. **(B)** DMPs' genomic locations for each methylation signature. The x-axis represents the number of DMPs. The left side shows the DMPs' distribution relative to genomic location; The right side shows the distribution relative to CpG islands. The length of different colored bars represents the proportion of DMPs. **(C)** Spearman correlation among DNA methylation signatures derived from TCGA samples. **(D)** Decomposition plots of hypermethylation and hypomethylation signatures, CC-Hyper-MS1-2 and CC-Hypo-MS1-2 from cell lines. DMPs’ locations in the CGI position are indicated by different colors.

**Fig S8**


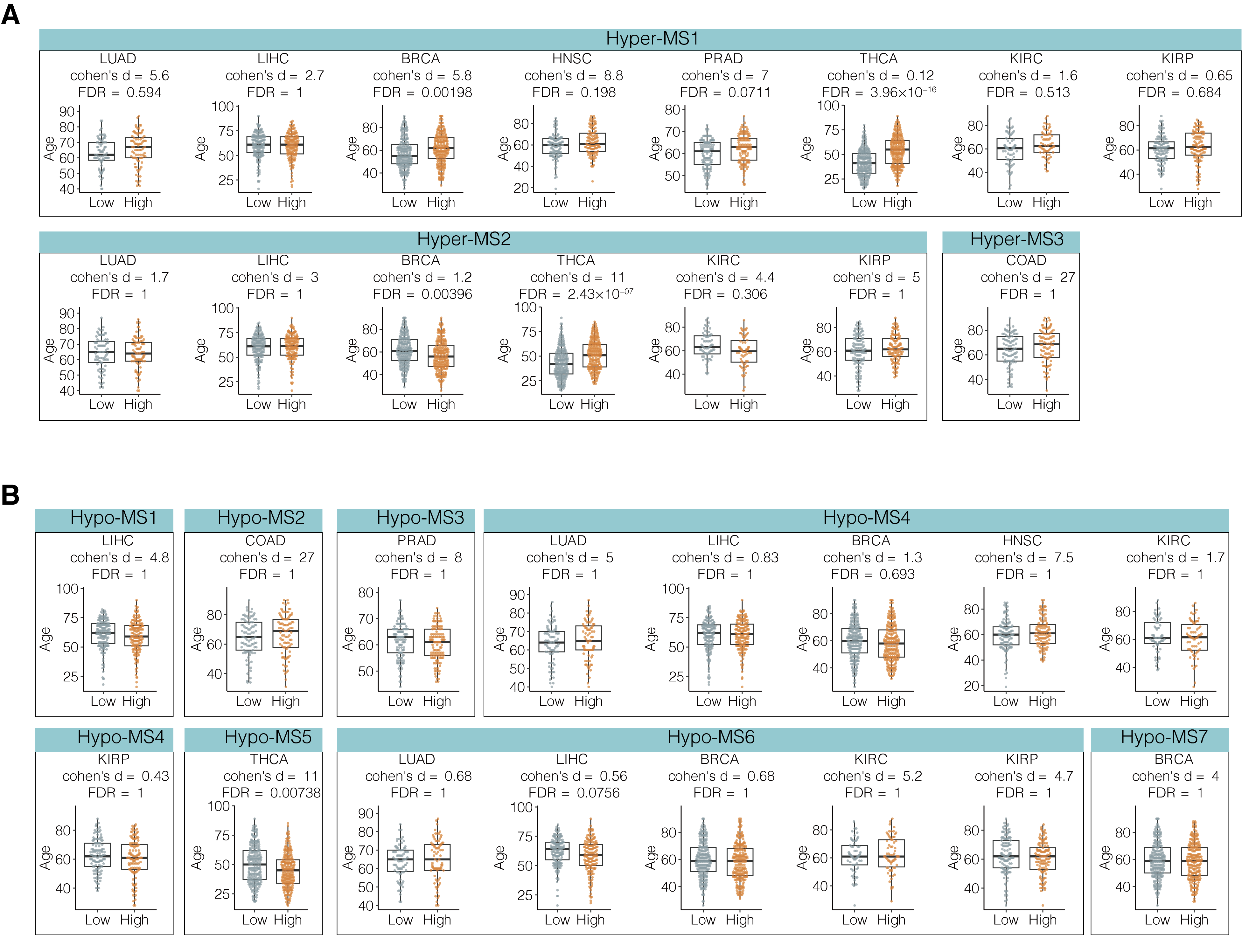


**Fig S8. Methylation signature activities association with age.**

**(A**) The relationship between patients' age and the activity of Hyper-MSs in individual cancer types, where the corresponding methylation signatures are present, with patients divided into two groups based on low and high signature activities (Wilcoxon's rank sum test). **(B)** The relationship between patients' age and the activity of Hypo-MSs in individual cancer types, where the corresponding methylation signatures are present, with patients divided into two groups based on low and high signature activities (Wilcoxon's rank sum test).

**Fig S9**


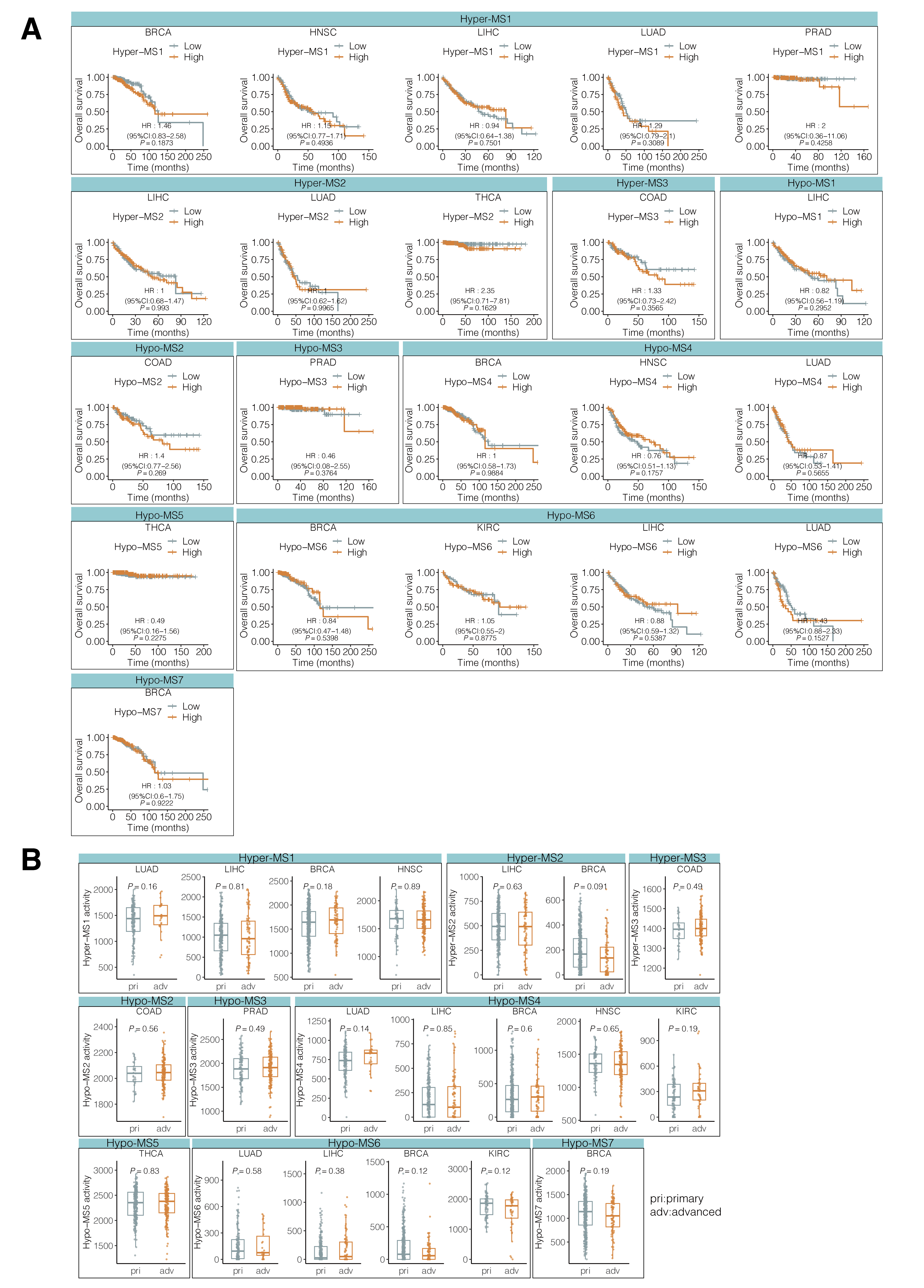


**Fig S9. Analysis of the correlations between overall survival, cancer stages and methylation signature activities.**

**(A)** Methylation signatures showed no significant association to the disease outcome the survival analysis. (**B**) The relationship between cancer stages and the activity of Hyper-MSs and Hypo-MSs in individual cancer types, where the corresponding methylation signatures are present. Patients are divided into two groups according to tumor stage (I, II as pri. and III, IV as adv.).

**Fig S10**


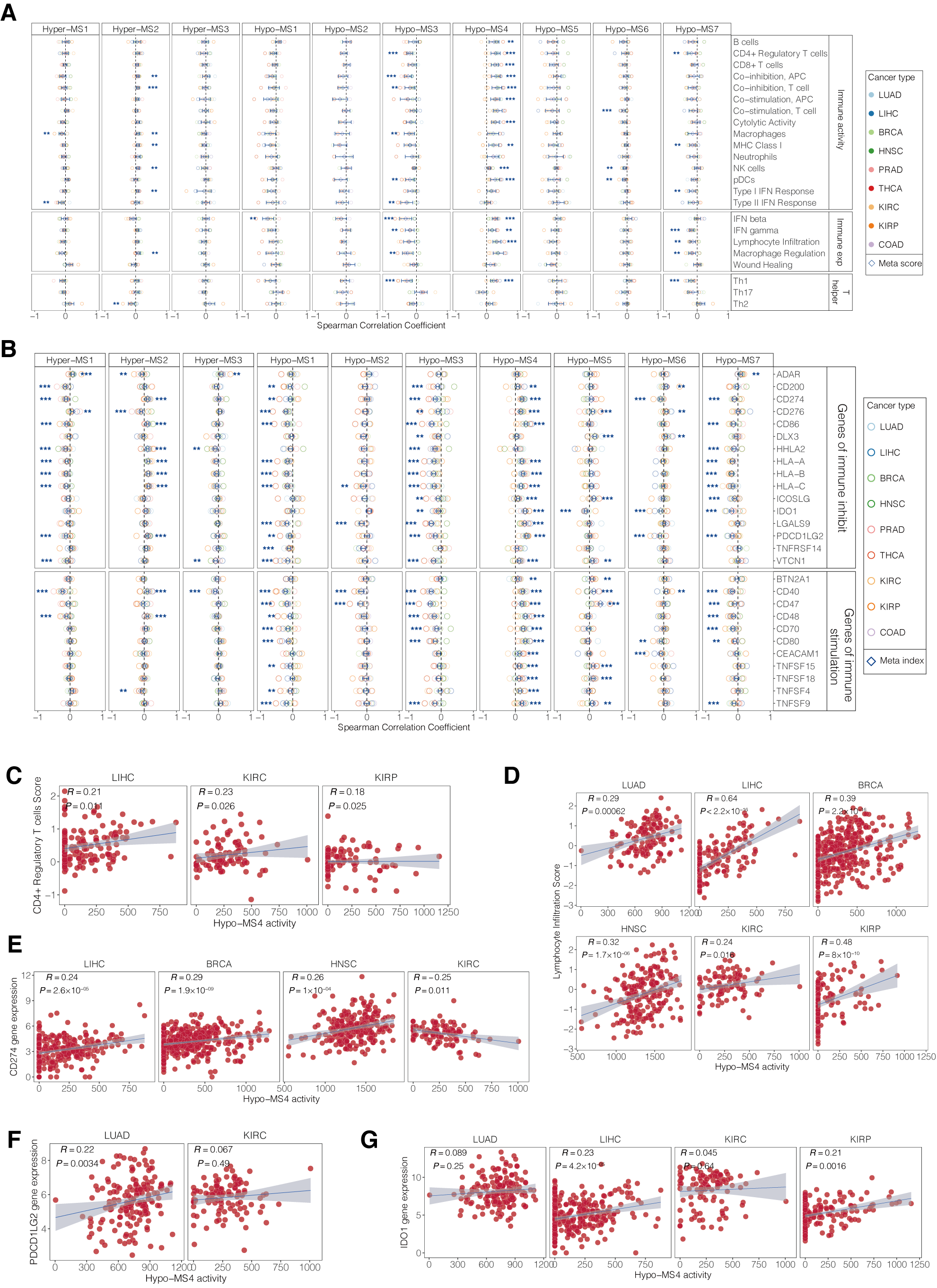


**Fig S10. The relationship between methylation signature activities and tumor immune microenvironment in cancers.**

**(A**) Forest plot of spearman correlation coefficients between tumor immunological features and methylation signatures at a pan-cancer level. Different colored circles represent different cancer types, and black lozenges represent meta-analysis scores. Asterisks indicate the significance of the meta coefficients, FDR < 0.05, *; FDR < 0.01, **; FDR < 0.001, ***. **(B)** Forest plot of Spearman correlation coefficients between the expression levels of immunological genes and methylation signatures at a pan-cancer level. Different colored circles represent different cancer types, and black lozenges represent meta-analysis scores. Asterisks represent the significance of similarity, FDR < 0.05, *; FDR < 0.01, **; FDR < 0.001, ***. **(C)** Correlation between the infiltrating CD4+ regulatory T cells score and Hypo-MS4 activity in LIHC, KIRC and KIRP. **(D)** Correlation between the Lymphocyte infiltration score and Hypo-MS4 activity in LUAD, LIHC, BRCA, HNSC, KIRC and KIRP. **(E)** Spearman correlation between the gene expression levels of CD274 and Hypo-MS4 activity in LIHC, BRCA, HNSC and KIRP. **(F)** Correlation between the gene expression level of PDCD1LG2 and Hypo-MS4 activity in LUAD and KIRC. **(G)** Correlation between the gene expression levels of IDO1 and Hypo-MS4 activity in LUAD, LIHC, KIRC and KIRP.

**Fig S11**


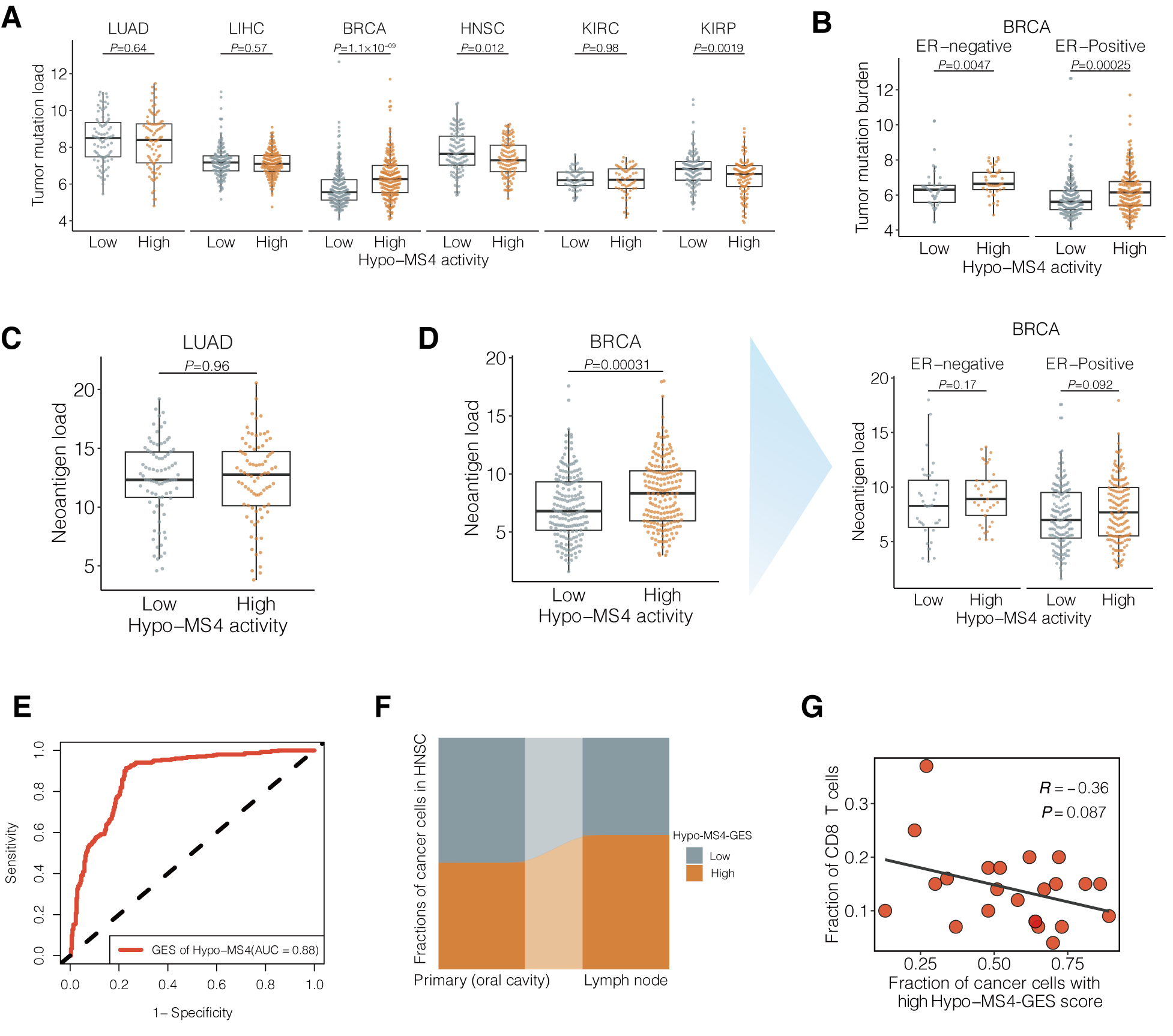


**Fig S11.** **The relationship between Tumor mutation burden, neoantigen load, tumor progression and Hypo-MS4 activity.**

**(A)** The differential high tumor mutation burden was compared between the low and Hypo-MS4 activity groups in individual cancer type where Hypo-MS4 is present. **(B)** The differential tumor mutation burden was compared between the low and high Hypo-MS4 activity groups with ER status. **(C)** The different neoantigen load was compared between the low and high Hypo-MS4 activity groups in LUAD. **(D)** The differential neoantigen load was compared between the low and high Hypo-MS4 activity groups in BRCA with ER-positive and ER-negative statuses. **(E)** ROC curve for predicting Hypo-MS4 activity using gene expression signature (GES) score of Hypo-MS4 in single-cell sequencing dataset of colorectal cancers. **(F)** Fractions of cancer cells with high and low Hypo-MS4-GES score (according to the mean GSE score of Hypo-MS4) in primary lesions and lymph node metastases from a single-cell RNA sequencing dataset of HNSC. **(G)** Pearson correlation between fractions of cancer cells with high Hypo-MS4-GES score and CD8+ T cells in a single-cell transcriptome sequencing of colorectal cancers.

**Fig S12**

**
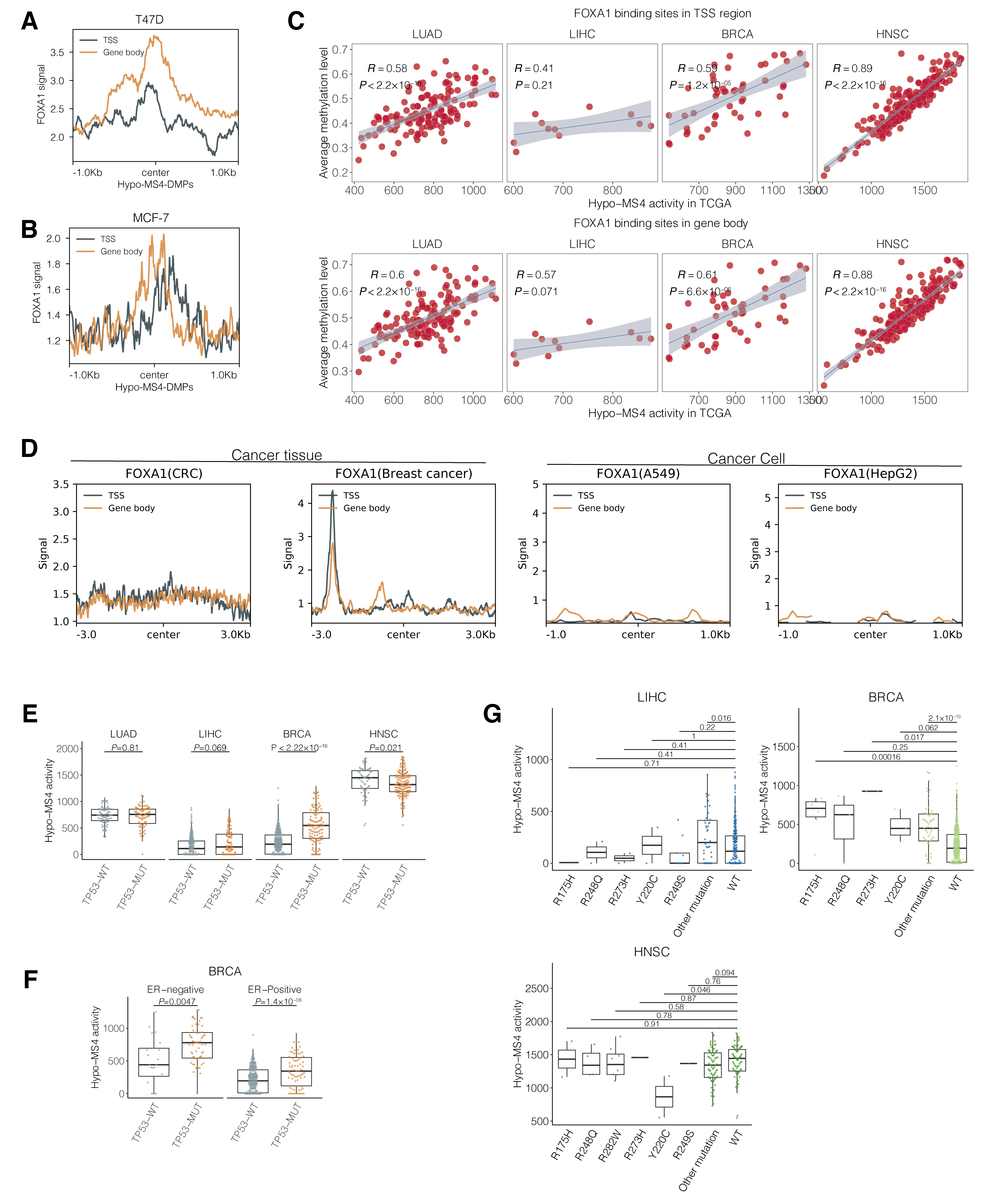
**

**Fig S12. Analysis of the correlations between deterministic genes and Hypo-MS4 activity.**

**(A-B)** ChIP-seq signal activity distribution of FOXA1 on Hypo-MS4-DMPs in cell lines (MCF-7 and T47D). **(C)** Spearman Correlation between the average methylation level of FOXA1 binding region and Hypo-MS4 activity in four cancer types from TCGA (Upper panel: FOXA1 binding sites in TSS region; Lower panel: FOXA1 binding sites in gene body). **(D)** FOXA1 binding affinity at Hypo-MS4-down-DMPs (as based on 1-β values). **(E)** Differential activities of Hypo-MS4 between WT and mutant TP53 patients in LUAD, LIHC, BRCA and HNSC. **(F)** The differential activities of Hypo-MS4 between WT and mutant TP53 patients with different ER status. **(G)** Differential activities of TP53 hotspots (Missense) in cancer types.

**Fig S13**


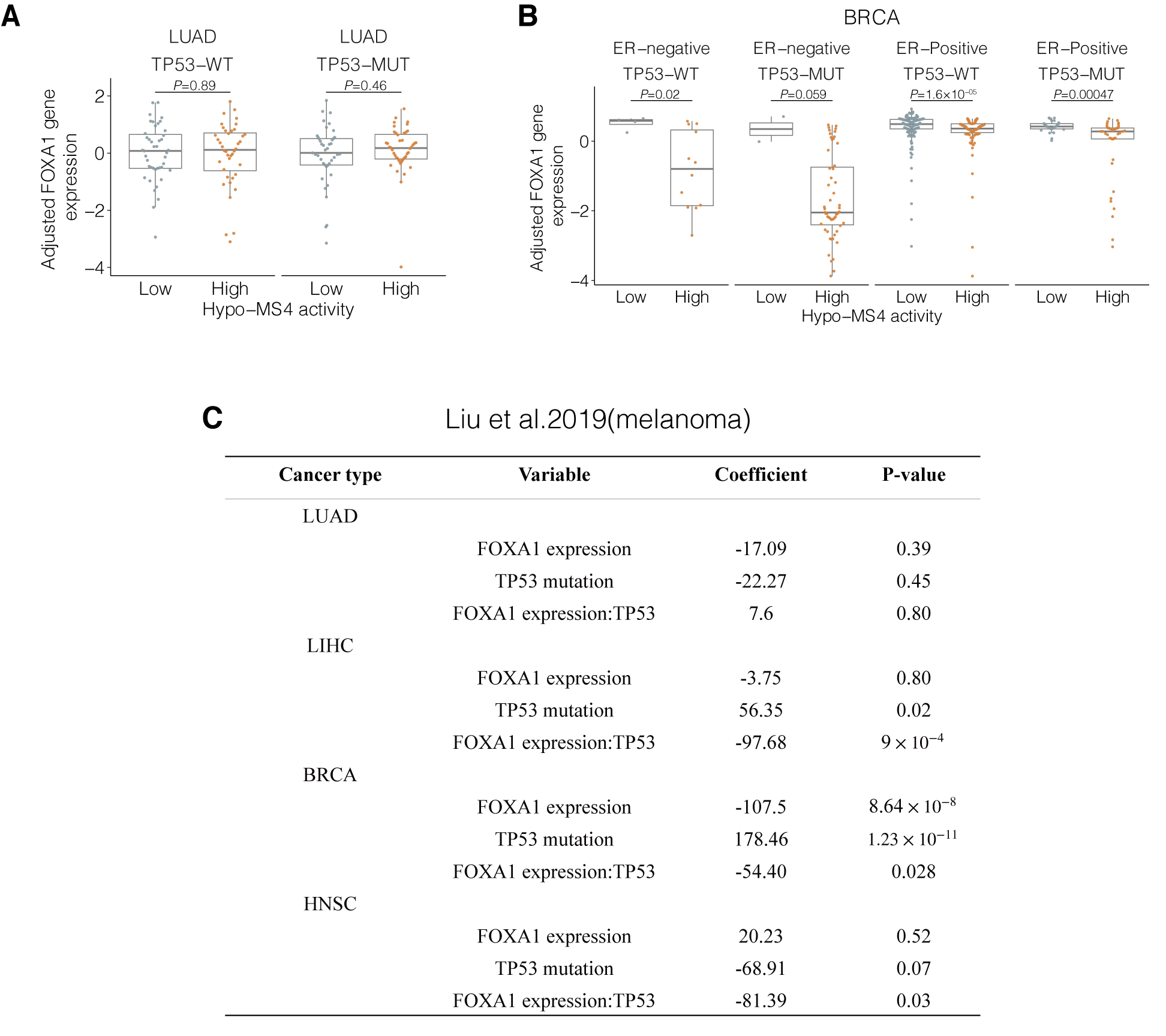


**Fig S13.** **Analysis of the intersection** **of deterministic genes and Hypo-MS4 activity.**

**(A)** Differential Hypo-MS4 activities between patients with low and high adjusted gene expression levels of FOXA1 in LUAD and patients further divided into TP53 WT and TP53-MUT groups. **(B)** Differential Hypo-MS4 activity in BRCA patients segmented by low and high adjusted FOXA1 gene expression levels, further stratified by TP53 mutation status in both ER-positive and ER-negative groups. **(C)** Multiple regression analysis delineates the interactive effects of FOXA1 gene expression and TP53 mutation status on the activity of the Hypo-MS4 methylation signature.

**Fig S14**


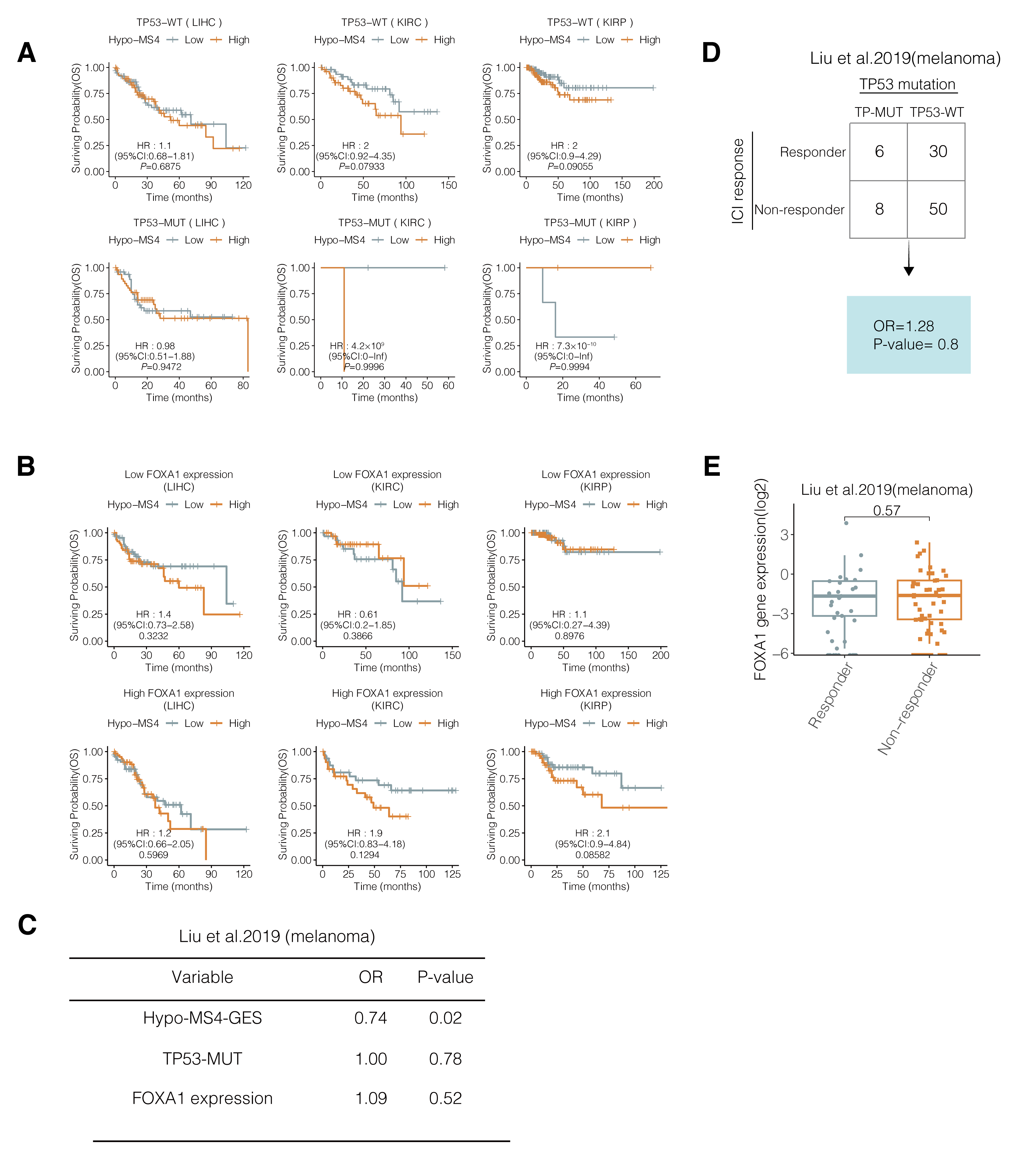


**Fig S14**. **Analysis of overall Survival and ICI response of Hypo-MS4 with deterministic genes status.**

**(A)** Survival analysis of Hypo-MS4 activity in groups of TP53-WT and TP53-MUT. **(B)** Survival analysis of Hypo-MS4 activity in groups of low and high FOXA1 gene expression. **(C)** Multiple regression analysis of Hypo-MS4-GES, FOXA1 gene expression and TP53 mutation with ICI response **(D)** The effect of mutation of TP53 on ICI response. **(E)** The differential FOXA1 gene expression between ICI response and non-response groups.
