# Supplementary material for: Conserved methylation signatures associate with the tumor immune microenvironment and immunotherapy response
Source: Genome Med. 2024 Apr 2;16:47. doi: 10.1186/s13073-024-01318-3 (PMC10985907; doi:10.1186/s13073-024-01318-3)
Supplement: Supplementary file 2 — Additional file 2: Table S1. Sample information. Table S2. Variation of DNA methylation signatures attributed to tissue origins. Table S3. Determinants of methylation signatures. [file 13073_2024_1318_MOESM2_ESM.docx]

**Supplementary Tables**

**Conserved methylation signatures associate with tumor immune microenvironment and immunotherapy response**

Qingqing Qin et.al.

**List of Supplementary Tables**

**Table S1:** Sample information.

**Table S2:** Variation of DNA methylation signatures attributed to tissue origins.

**Table S3:** Determinants of methylation signatures.

**Table S1: Sample information**

| **Cancer type** | **No. of tumor samples** | **No. of paired tumor samples used to derive DMPs** | **No. of tumor samples purity > 0.6 used to derive signatures** |
| --- | --- | --- | --- |
| **BRCA** | 785 | 98 | 460 |
| **COAD** | 313 | 38 | 197 |
| **HNSC** | 528 | 50 | 234 |
| **KIRC** | 319 | 160 | 132 |
| **KIRP** | 275 | 45 | 244 |
| **THCA** | 507 | 56 | 458 |
| **PRAD** | 498 | 50 | 259 |
| **LIHC** | 377 | 50 | 321 |

**Table S2: Variation of DNA methylation signatures attributed to tissue origins.**

| Signature | Variations attributed to tissue origins |
| --- | --- |
| Hyper-MS1 | 0.79 |
| Hyper-MS2 | 0.80 |
| Hyper-MS3 | 0.99 |
| Hypo-MS1 | 0.80 |
| Hypo-MS2 | 0.99 |
| Hypo-MS3 | 0.92 |
| Hypo-MS4 | 0.78 |
| Hypo-MS5 | 0.97 |
| Hypo-MS6 | 0.86 |
| Hypo-MS7 | 0.86 |

**Table S3: Determinants of methylation signatures.**

| Signature | No. of gene mutations (SNV) | No. of gene mutations (Frame shift) |
| --- | --- | --- |
| Hyper-MS1 | 42 | 62 |
| Hyper-MS2 | 4066 | 83 |
| Hyper-MS3 | 4335 | 84 |
| Hypo-MS1 | 85 | 57 |
| Hypo-MS2 | 3898 | 89 |
| Hypo-MS3 | 1071 | 64 |
| Hypo-MS4 | 451 | 63 |
| Hypo-MS5 | 1973 | 70 |
| Hypo-MS6 | 1934 | 70 |
| Hypo-MS7 | 330 | 71 |
